# Routing Nanomolar Protein Cargoes to Lipid Raft‐Mediated/Caveolar Endocytosis through a Ganglioside GM1‐Specific Recognition Tag

**DOI:** 10.1002/advs.201902621

**Published:** 2020-01-09

**Authors:** Norbert Imre, Anasztázia Hetényi, Enikő Szabó, Brigitta Bodnár, Abel Szkalisity, Ilona Gróf, Alexandra Bocsik, Mária A. Deli, Peter Horvath, Ágnes Czibula, Éva Monostori, Tamás A. Martinek

**Affiliations:** ^1^ Department of Medical Chemistry University of Szeged Dóm tér 8 Szeged HU‐6720 Hungary; ^2^ Institute of Genetics Biological Research Center (BRC) Temesvári krt. 62 Szeged HU‐6726 Hungary; ^3^ MTA‐SZTE Biomimetic Systems Research Group University of Szeged Dóm tér 8 Szeged HU‐6720 Hungary; ^4^ Synthetic and Systems Biology Unit Biological Research Center (BRC) Temesvári krt. 62 Szeged HU‐6726 Hungary; ^5^ Institute of Biophysics Biological Research Center (BRC) Temesvári krt. 62 Szeged HU‐6726 Hungary; ^6^ Doctoral School of Biology University of Szeged Dugonics tér 13 Szeged HU‐6720 Hungary; ^7^ Department of Cell Biology and Molecular Medicine University of Szeged Somogyi u. 4 Szeged HU‐6720 Hungary; ^8^ Institute for Molecular Medicine Finland University of Helsinki Tukholmankatu 8 Helsinki FI‐00014 Finland

**Keywords:** cell delivery, endocytosis, glycan, immunoglobulin, peptides

## Abstract

There is a pressing need to develop ways to deliver therapeutic macromolecules to their intracellular targets. Certain viral and bacterial proteins are readily internalized in functional form through lipid raft‐mediated/caveolar endocytosis, but mimicking this process with protein cargoes at therapeutically relevant concentrations is a great challenge. Targeting ganglioside GM1 in the caveolar pits triggers endocytosis. A pentapeptide sequence WYKYW is presented, which specifically captures the glycan moiety of GM1 (*K*_D_ = 24 nm). The WYKYW‐tag facilitates the GM1‐dependent endocytosis of proteins in which the cargo‐loaded caveosomes do not fuse with lysosomes. A structurally intact immunoglobulin G complex (580 kDa) is successfully delivered into live HeLa cells at extracellular concentrations ranging from 20 to 160 nm, and escape of the cargo proteins to the cytosol is observed. The short peptidic WYKYW‐tag is an advantageous endocytosis routing sequence for lipid raft‐mediated/caveolar cell delivery of therapeutic macromolecules, especially for cancer cells that overexpress GM1.

## Graphical abstract

Targeting ganglioside monosialotetrahexosylganglioside (GM1) in the lipid rafts with specific binding sequences can induce caveolar endocytosis of macromolecules, a route that avoids lysosomal entrapment. A pentapeptide sequence that can deliver a 580 kDa structurally functional immunoglobulin G complex at nanomolar concentrations is presented, opening up new ways for intracellular antibody delivery.

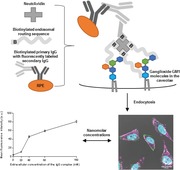

## 1 Introduction

The mammalian cell membrane is a major obstacle in drug development because it represents a barrier that is mostly impermeable to extracellular proteins that can potentially act as specific, efficient, and tolerable drugs.^1^, ^2^ Translocation of macromolecular cargo is possible through endocytosis, and lipid raft‐mediated/caveolar endocytosis^3^, ^4^ is exploited by viruses (simian virus 40 and murine polyomavirus,^5^ and echovirus 1^6^), bacterial toxins (cholera and tetanus),^7^ and endogenous proteins^8^ to deliver macromolecules in their functional form without degradation. Targeting this clathrin‐independent pathway is an especially attracting strategy because direct escape from the internalized compartments is possible before moving to other cellular locations as exemplified by echovirus 1.^6^ A frequent feature of these natural cargoes is theability to bind and cluster mono‐, di‐, and trisialotetrahexosylgangliosides (GM1, GD1a, GT1b) during their attachment to caveolar pits, which induces endocytosis.^9^, ^10^ Entry through ganglioside recognition is an attractive strategy because progression of the internalized caveolae to endosomes and later to lysosomes is slow or absent, allowing time for delivery of the contents to cell organelles and the cytosol^11^ or transcytosis.^12^, ^13^ Selection of the ganglioside‐mediated pathway addresses many of the challenges associated with the endocytic delivery of therapeutic macromolecules^14^ by allowing carrier‐triggered internalization at low concentrations and avoiding endosomal entrapment. For this reason, there is increasing interest in reading the glycan code through which gangliosides trigger lipid raft‐mediated/caveolar endocytosis.^15^, ^16^ The referenced studies identified peptide sequences (13 and 16 residues) that bind to various gangliosides. However, the high‐affinity (low nanomolar) molecular recognition of gangliosides that is necessary for therapeutic applications is still a great challenge. Specific and high‐affinity targeting of ganglioside GM1 is especially sought because GM1 is expressed in many mammalian cell types, including endothelial cells, and is overexpressed in cancer cells.^17^, ^18^ Because therapeutic protein levels in the extracellular fluid do not exceed the nanomolar range (clinical protocols yield 100–500 nm),^19^ high‐affinity target–carrier interaction is necessary to create a cell surface enrichment that facilitates sufficient material flux in potential clinical applications. State‐of‐the‐art methods for this include the use of multimeric peptides,^20^ supercharged protein carriers^21^ and complexes of such carriers with cationic lipids^22^ that target generic negative patches of the membrane, which then translocate intact functional macromolecules at nanomolar concentrations.

Our goal in this work was to achieve nanomolar delivery of large proteins (e.g., IgG) via lipid raft‐mediated/caveolar endocytosis. We set out to find a short peptide tag, a non‐toxic minimal motif that is able to specifically route its macromolecular cargo to an entry pathway that mimics the ganglioside‐mediated internalization of viruses and bacterial toxins through the major lipid raft/caveolar component GM1. By focusing on a structurally well‐defined receptor and conducting a thorough biophysical characterization of the interaction, we aimed to open the way to structure‐based design, which is rare in protein delivery approaches.^23^, ^24^ Here, we show that a minimal palindromic pentapeptide sequence (WYKYW) specifically binds the carbohydrate moiety of GM1 with low nanomolar affinity. Direct attachment of this short endocytosis routing tag to large proteins facilitated lipid raft‐mediated/caveolar endocytosis of the macromolecules at therapeutically relevant nanomolar concentrations and avoided endosomal entrapment and lysosomal degradation.

## 2 Results

### 2.1 Pentapeptide WYKYW Binds Ganglioside GM1 with High Affinity and Specificity

**Figure 1:**
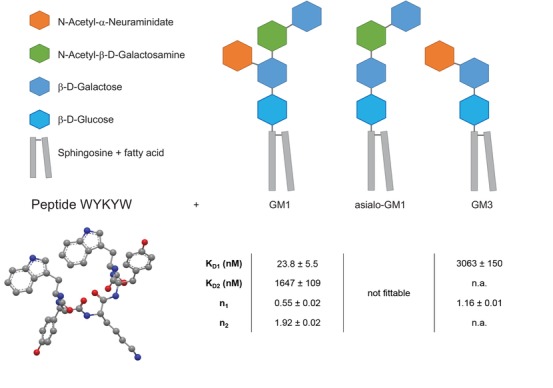
Structure of the targeted ganglioside GM1 and its truncated derivatives and sequence, and structure of the lead peptide sequence WYKYW. Binding affinities (*K*_D_) and stoichiometries (*n*) for ganglioside–WYKYW interactions were measured by ITC. Parameters were obtained by nonlinear least squares fitting against the two‐independent‐binding‐site and the single‐binding‐site models for GM1 and GM3, respectively. For asialo‐GM1, the ITC enthalpogram did not exhibit fittable features.

The lead molecule WYKYW (**Figure** **1**) was first identified by Gabius and coworkers^25^, ^26^ as a member of the Tyr‐Xxx‐Tyr‐containing pentapeptide family, which was observed to decouple galectin‐1 and proteoglycan interactions.^27^ Subsequently, we showed that the mechanism of inhibition of the proteoglycan–galectin‐1 interaction is a competitive binding of the peptide at the glycan moiety of asialofetuin.^28^ Knowing that galectin‐1 binds to ganglioside GM1,^29^ we hypothesized that WYKYW also interacts with the extracellular glycan moiety of GM1. To that end, we synthesized the peptide and measured its affinity and specificity for different gangliosides using isothermal titration calorimetry (ITC) (Figure 1; Figure S1a, Supporting Information) and NMR (Figure S2–S4, Supporting Information).

**Figure 2:**
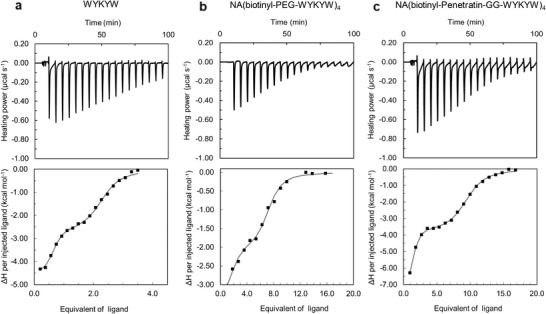
ITC enthalpograms for a) WYKYW, b) NA(biotinyl‐PEG‐WYKYW)_4_, and c) NA(biotinyl‐Penetratin‐GG‐WYKYW)_4_. Titrations were conducted with GM1:DPC 1:5 bicelles (solid square), and nonlinear least squares fitting was performed against the two‐independent‐binding‐site model (solid lines).

Two‐stage interaction with GM1 was observed (**Figure** **2**a) in which the first binding step displayed low nanomolar affinity with a GM1:WYKYW stoichiometry of 1:2. The second stage was a micromolar interaction. In the control experiment with pure n‐dodecylphosphocholine (DPC), no interaction was found. Removal of the sialyl group from GM1 (asialo‐GM1) was detrimental to binding. For ganglioside GM3, which lacks the terminal β‐D‐Gal(1→3)GalNAc, only a weak interaction was observed (Figure S1, Supporting Information). NMR spectroscopy confirmed the ITC results. Effective NMR signal attenuation and broadening occurred upon mixing WYKYW with GM1:DPC bicelles^30^ (Figure S2, Supporting Information) as a result of the strong interaction. GM3:DPC mixture yielded partial line broadening in the ^1^H NMR spectrum, and saturation transfer difference (STD) signals were observed (Figure S3, Supporting Information), indicating weak binding involving an elevated exchange rate. Asialo‐GM1:DPC bicelles and pure DPC micelles left the NMR resonances of WYKYW intact (Figure S4, Supporting Information), and STD could not be detected. ITC and NMR measurements revealed that WYKYW binds only GM1 with low nanomolar affinity. We note that fluorescein tagging in the proximity of sequence WYKYW could significantly decrease the binding affinity to GM1 (Figure S5, Supporting Information); therefore, it is not a preferred setup.

### 2.2 The WYKYW‐Tag Triggers Lipid Raft‐Mediated/Caveolar Endocytosis of Protein Cargo at Nanomolar Concentrations

**Figure 3:**
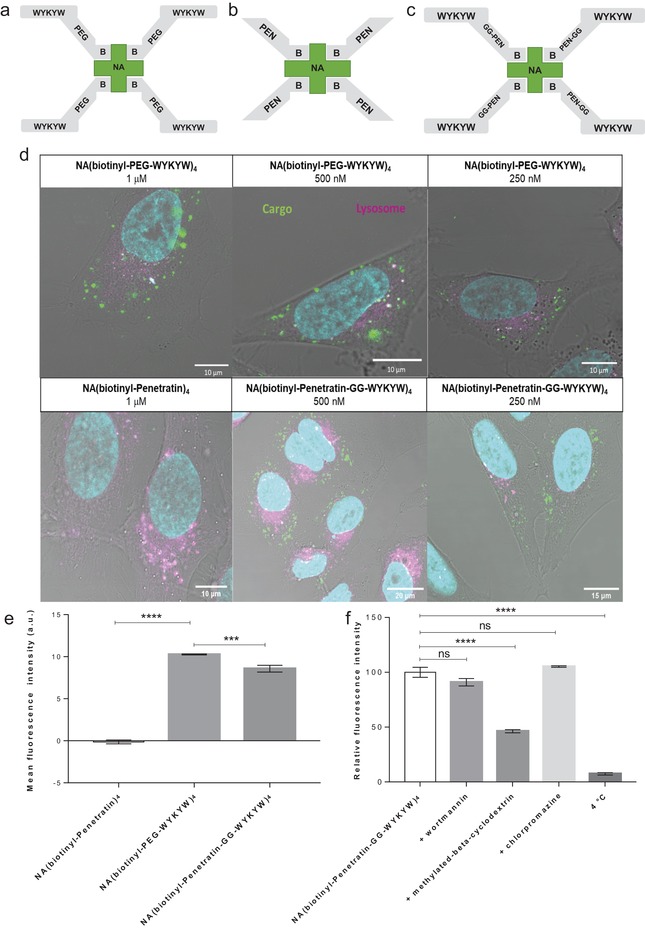
Schematic representation of a) NA(biotinyl‐PEG‐WYKYW)_4_, b) NA(biotinyl‐Penetratin)_4_, and c) NA(biotinyl‐Penetratin‐GG‐WYKYW)_4_. d) Internalization of the constructs at different concentrations by HeLa cells after 6 h as determined by live confocal laser scanning microscopy. FITC‐labeled NA is shown in green, Hoechst 33342‐stained nuclei are shown in cyan, and LysoTracker Red‐stained lysosomes are shown in magenta. e) Internalization of the constructs at 1 µm by HeLa cells after 1 h as determined by flow cytometry. f) Influence of endocytosis inhibitors on cellular uptake as determined by flow cytometry. HeLa cells were preincubated with the inhibitors wortmannin (W), chlorpromazine (CP), or β‐methyl‐cyclodextrin (BMCD) at 37 °C for 30 or 60 min and subsequently incubated with NA(biotinyl‐Penetratin‐GG‐WYKYW)_4_ at 37 °C for 60 min. A control experiment was also performed at 4 °C. Each data point depicts the mean of three measurements; the error bars show the standard error of the mean. Statistical analysis was performed using one‐way analysis of variance (ANOVA) with post hoc Tukey honestly significant difference test. *p < 0.1; **p < 0.01; ***p < 0.001; ****p < 0.0001.

In the next step, we tested the hypothesis that the high‐affinity WYKYW–GM1 interaction induces endocytosis when the carrier tag is attached to a protein. We selected FITC‐labeled NeutrAvidin (NA) as the model cargo; we tagged it with a biotinyl‐PEG‐WYKYW conjugate (**Figure** **3**), where PEG designates a trimeric linker obtained by coupling of 8‐amino‐3,6‐dioxa‐octyl)succinamic acid monomer. NA binds four biotinylated sequences, yielding the tetravalent protein construct NA(biotinyl‐PEG‐WYKYW)_4_ (Figure 3a). To assess the performance and the endocytosis‐inducing efficiency of WYKYW relative to a reference cell‐penetrating sequence, FITC‐NA was tagged with biotinyl‐Penetratin, yielding NA(biotinyl‐Penetratin)_4_ (Figure 3b). To measure the additive and synergistic effects of WYKYW and Penetratin, a biotinyl‐Penetratin‐GG‐WYKYW chimera (NA(biotinyl‐Penetratin‐GG‐WYKYW)_4_) was also used (Figure 3c). ITC measurements confirmed that NA(biotinyl‐PEG‐WYKYW)_4_ and NA(biotinyl‐Penetratin‐GG‐WYKYW)_4_ bound GM1 with *K*_D_s of 14.5 ± 1.7 and 20.8 ± 2.7 nm, respectively (Figure 2b,c). The stoichiometry of the interaction was 1:1 under conditions of excess of the tagged protein. NA(biotinyl‐Penetratin)_4_ displayed no affinity for GM1.

Live confocal laser‐scanning microscopy (CLSM) experiments were conducted to test the entry of the WYKYW‐containing conjugates into cells (Figure 3d). Effective uptake of both NA(biotinyl‐PEG‐WYKYW)_4_ and NA(biotinyl‐Penetratin‐GG‐WYKYW)_4_ was observed over an extracellular cargo concentration range of 250–1000 nm. Surprisingly, NA(biotinyl‐Penetratin)_4_ did not enter the cells under these conditions, as can be seen from the CLSM images.

Cell internalization of the carrier‐cargo constructs was quantified by fluorescence‐activated cell sorting (FACS) measurements at cargo concentrations of 1 µm (Figure 3e) with trypan blue as an extracellular fluorescence quencher. The levels of uptake of NA(biotinyl‐PEG‐WYKYW)_4_ and NA(biotinyl‐Penetratin‐GG‐WYKYW)_4_ were similar, while the Penetratin‐tagged control NA(biotinyl‐Penetratin)_4_ was not delivered into the cells. The CLSM and FACS results strongly suggest that the GM1 recognition sequence WYKYW was able to trigger endocytosis when attached to the model protein through a linker. In contrast, the Penetratin‐tag did not induce endocytosis of the cargo under these conditions (Figure 3d,e). The presence of Penetratin in the WYKYW‐containing carrier sequence reduced the cell uptake efficiency relative to the PEG linker derivative (absence of additive or synergistic effects), indicating that WYKYW has a reliable endocytosis‐inducing effect that is independent of the linker chemistry.

Simultaneous staining of lysosomes showed no colocalization of lysosomes with the carrier–cargo complex even after 6 h (Figure 3d), indicating the ability of WYKYW to successfully target the lipid raft‐mediated/caveolar endocytosis pathway. To gain further support for the selectivity of the cell entry mechanism, we performed endocytosis inhibition experiments with NA(biotinyl‐Penetratin‐GG‐WYKYW)_4_ in HeLa cells. The internalization of the complex could be blocked at 4 °C, proving that the translocation was energy‐dependent (Figure 3f). After pretreatment of the cells with various inhibitors of endocytosis, we observed that methylated β‐cyclodextrin (BMCD), a known lipid raft inhibitor, significantly decreased the entry of the complex, while wortmannin and chlorpromazine had no significant effects. This confirmed the lipid raft‐mediated/caveolar endocytosis pathway (Figure 3f), consistent with the fact that GM1 is localized in lipid rafts and caveolae.^31^ To gain additional supporting evidence for the lipid raft‐mediated pathway, we carried out a colocalization experiment with the carrier–cargo complex tagged with Alexa Fluor 647 on the secondary antibody and FITC‐labeled cholera toxin B subunit. Cholera toxin has been reported to enter cells through GM1 binding and lipid raft‐mediated way.^7^ We found srtrong correlation between the signals observed for cholera toxin and the carrier–cargo complex (Figure S6, Supporting Information), indicating that the carrier–cargo complex entered the cells through lipid raft‐mediated mechanism.

### 2.3 A Single WYKYW‐Tag Is Sufficient to Trigger Endocytosis through Ganglioside GM1 Binding

**Figure 4:**
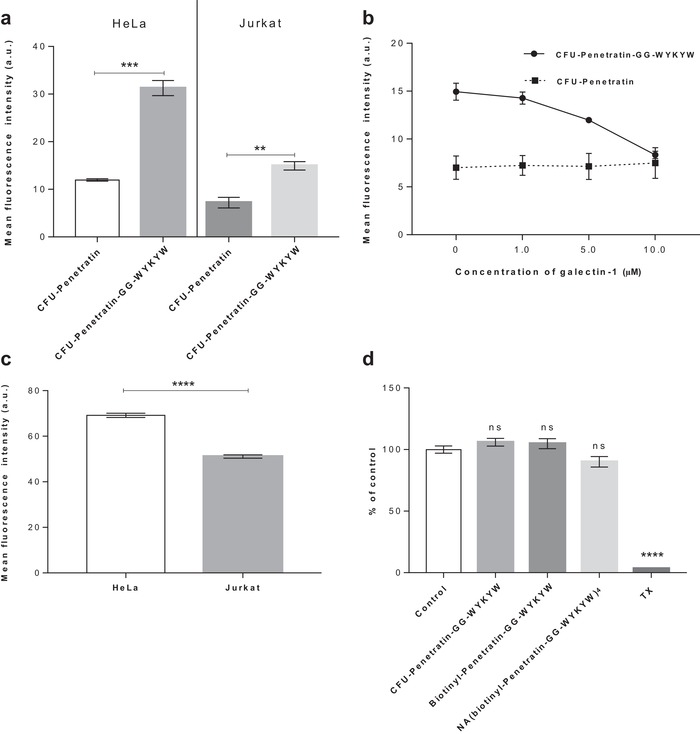
a) Uptake of fluorescently labeled sequences at 1 µm by HeLa and Jurkat cells after 1 h. b) Uptake of CFU‐Penetratin and CFU‐Penetratin‐GG‐WYKYW at 1 µm in competition with galectin‐1 at 0–10 µm. c) Cell surface expression of GM1 in HeLa and Jurkat cells measured by FITC‐cholera toxin staining. d) Cytotoxicity of CFU‐Penetratin‐GG‐WYKYW, biotinyl‐Penetratin‐GG‐WYKYW, and NA(biotinyl‐Penetratin‐GG‐WYKYW)_4_ at 10 µm to HeLa cells after 24 h as determined by bioimpedance measurements. Triton X‐100 was used as a toxicity control. Each data point represents the mean of three measurements, and the error bars show the standard error of the mean. The unpaired Student's *t*‐test was used in the statistical analysis of the data shown in panels (a) and (c): *p < 0.05; **p < 0.01; ***p < 0.001; ****p < 0.0001. One‐way analysis of variance (ANOVA) with post hoc Tukey honestly significant difference test was used in the statistical analysis of the data shown in panel (d): *p < 0.1; **p < 0.01; ***p < 0.001; ****p < 0.0001.

Helenius and Pelkmans pointed out that multivalent binding/clustering of ganglioside GM1 is necessary to trigger lipid raft‐mediated/caveolar endocytosis,^9^ and the tetravalent nature of our model carrier–cargo complex is consistent with this observation. On the other hand, the number of copies of the carrier sequence required for endocytosis can be crucial, especially if the carrier–cargo complex is produced by recombinant synthesis. To that end, we tested the uptake of the monovalent CFU‐Penetratin‐GG‐WYKYW conjugate, which displayed a *K*_D_ of 141 ± 45 nm toward GM1. CFU‐Penetratin, which has no GM1 binding affinity, was utilized as a control. The cellular uptake of these sequences by human HeLa and Jurkat cell lines was measured by FACS (**Figure** **4**a) with trypan blue as an extracellular fluorescence quencher. The carriers were applied at a concentration of 1 µm, which is at least one order of magnitude lower than the optimum concentration utilized for penetratin,^32^ but we observed cell penetration of the stand‐alone CFU‐Penetratin without the protein cargo. The CFU‐Penetratin‐GG‐WYKYW complex displayed three‐ and twofold increases in cell penetration efficiency in the HeLa and Jurkat cell lines, respectively, compared with that obtained for CFU‐Penetratin without macromolecular cargo. The amount of intracellular cargo was twice as large in HeLa cells as in Jurkat cells. We hypothesized that the cell‐dependent performance of WYKYW was related to the cell surface expression level of GM1. Binding experiments performed with the GM1 binder cholera toxin at 4 °C (which prevents endocytosis) revealed that HeLa cells expressed a higher level of GM1 than Jurkat cells (Figure 4c). Next, the direct GM1‐dependence of the endocytosis was tested in a competition experiment in which galectin‐1, which binds terminal digalactosides with micromolar affinity,^33^ was applied as an inhibitor.

At a concentration of 10 µm, galectin‐1 decreased the uptake of CFU‐Penetratin‐GG‐WYKYW to the base level displayed by CFU‐Penetratin (Figure 4b). These findings suggest that a single WYKYW segment is sufficient to trigger endocytosis through GM1 binding, leading to efficient delivery. The amount of internalized cargo correlated with the cell surface expression level of GM1 (Figure 4c). The WYKYW‐tag showed a number of advantageous properties, and we observed no signs of cytotoxicity during the cell‐based experiments. The possible cytotoxicity of CFU‐Penetratin‐GG‐WYKYW was also tested at higher concentrations, and it was not toxic to HeLa cells at concentrations of up to 10 µm (Figure 4d), rendering it a safe candidate for further experiments.

### 2.4 The WYKYW‐Tag Facilitates Intracellular Delivery of a Large Antibody Complex at Therapeutically Relevant Nanomolar Concentrations

**Figure 5:**
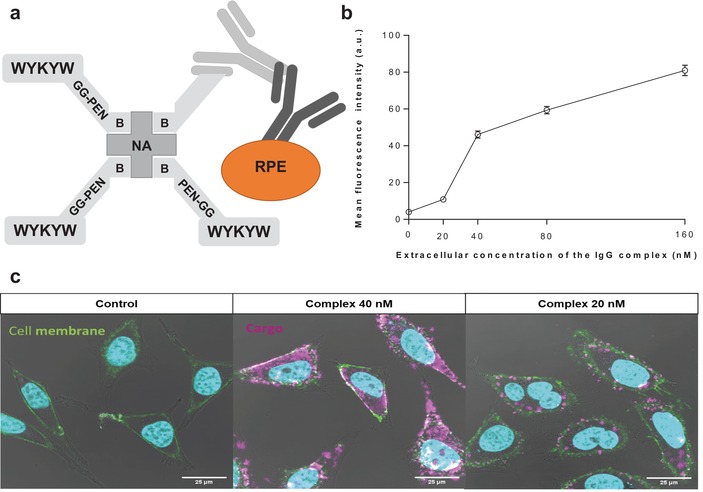
a) Schematic representation of the bottom‐up designed modular carrier–hub–antibody cargo–secondary antibody–r‐phycoerythrin construct. b) Artificial intelligence‐aided quantitative analysis of the live CLSM images. HeLa cells were incubated for 6 h with various concentrations of the IgG complex; at least 75 representative cells were then analyzed at each concentration. The intensity value obtained for the control sample is indicated at zero concentration. c) Delivery of the IgG complex into HeLa cells at various concentrations after 3 h. R‐phycoerythrin‐conjugated secondary antibody is indicated in magenta; green staining defines cell membranes (WGA‐FITC). Nuclei are indicated in cyan. Control cells were treated with r‐phycoerythrin‐labeled secondary antibody at 160 nm for 3 h.

In the next step, we investigated whether the WYKYW‐tag is able to induce endocytosis of a large protein cargo belonging to the therapeutically relevant family of immunoglobulins. We designed a 580‐kDa construct that contains NA as a connection hub, the WYKYW‐containing carrier tag biotinyl‐PEG‐WYKYW or biotinyl‐Penetratin‐GG‐WYKYW, a biotinylated primary immunoglobulin G, and a secondary antibody labeled with R‐phycoerythrin (**Figure** **5**a).

HeLa cells were incubated with the large carrier–cargo complex for 3 hours at various extracellular concentrations. We set out to test the possible lower limit of the affinity‐driven endocytic material flux; therefore, we performed the experiments at concentrations close to the measured *K*_D_ for the carrier–hub complex (21 nm). To eliminate the surface‐bound fraction, the samples were subjected to thorough washing with unlabeled NA(biotinyl‐Penetratin‐GG‐WYKYW)_4_. The carrier–IgG complex with the biotinyl‐Penetratin‐GG‐WYKYW conjugate was internalized over the concentration range of 20–160 nm (Figure 5; Figure S7 and S8, Supporting Information).

Unfortunately, protein precipitation was observed during sample preparation with biotinyl‐PEG‐WYKYW. This finding suggests that the linker region could function as a customizable segment to stabilize the carrier–cargo complex in solution. Importantly, diffuse fluorescence was observed between the caveosomes throughout the cytoplasm when the complex was applied at concentrations above 40 nm, indicating the ability of the system to escape the internalized compartments.^34^ Visual inspection suggested, however, that the amount of internalized cargo decreased at 20 nm. Artificial intelligence‐aided quantitative analysis of the CLSM images was conducted (Figure 5b; Figure S7, Supporting Information). The results confirmed that the complex containing the GM1 recognition segment WYKYW was a robust carrier agent that triggered endocytosis and translocation of the 580 kDa cargo at extracellular concentrations corresponding to those in the lower range of therapeutic protocols (ca. 100 nm). The decrease in the relative amount of translocated cargo at approximately 20 nm was confirmed by the analysis, and that observation places the lower performance limit close to the *K*_D_ value of the WYKYW–GM1 interaction. This phenomenon, which can be explained by the law of mass action, supports the idea that endocytosis of the cargo was driven by the GM1 affinity tag. The intense intracellular fluorescence emission of r‐phycoerythrin was a telltale sign of the functional protein. To test a possible degradation of the IgG components between the carrier and the r‐phycoerythrin, a colocalization experiment was run with FITS‐NA in the carrier and an Alexa Fluor 647‐tagged secondary antibody in the cargo. We found very good spatial correlation between the two fluorescent signals (Figure S9, Supporting Information), which strongly supported that the molecular recognition between the primary and the secondary antibody was functional. As an additional test for the structural integrity of the primary antibody, HeLa cells were treated with a carrier–cargo complex containing only a primary anti‐galectin‐1 antibody attached to the carrier, and the internalized IgG component was visualized by using Atto 488‐labeled galectin‐1 after fixation and permeabilization of the cells (Figure S10, Supporting Information). The control cells untreated with the carrier–cargo complex did not display fluorescence, while the internalized antibody bound the fluorescent galectin‐1 in the treated cells and fluorescence was observed. This finding strongly suggested that the Fv region the primary antibody was structurally intact.

## 3 Discussion

Mammalian cells exert strict control over macromolecular traffic through the cell membrane to cellular compartments. Lipid raft‐mediated/caveolar endocytosis is the most promising method for delivering cargo proteins in their functional form, as exemplified by viruses and bacterial toxins.^15^ Our concept was therefore to steer the macromolecular cargo toward lipid raft‐mediated/caveolar endocytosis and to avoid the clathrin‐mediated and macropinocytosis pathways. We focused on the initial molecular recognition events that occur at endocytic membrane pits because this facilitated both the selection of the mechanism and the effective enrichment of the low‐concentration cargo at the entry point. We found that the WYKYW‐tag binds the glycan moiety of the caveolar receptor GM1 with high affinity. The lack of strong interactions with GM3 and asialo‐GM1 indicated selective behavior. We concluded that both the sialyl group and the terminal *N*‐Ac‐digalactoside in GM1 are essential structural features for the low nanomolar binding.

An important feature of the WYKYW‐tag is that it can effectively route the macromolecular cargo to the desired lipid raft‐mediated/caveolar endocytosis entry point, and it can induce the pinch‐off process even when attached to a large cargo‐containing IgG proteins. Based on the specific affinity‐based directing effect of WYKYW, we define the term “endocytosis routing sequence.” Although multivalent binding of caveolar GM1 has been reported to be necessary to trigger endocytosis, we found that incorporation of a single WYKYW segment into the chain is sufficient to initiate internalization through GM1 binding. We note that galectin‐1 acted as a competitive inhibitor of the GM1–WYKYW interaction but this occurred only at concentrations orders of magnitude higher than the endogenous in vivo serum galectin‐1 level of 100 ng mL^–1^ (6.7 nm).^35^ Based on this observation, the risk of potential in vivo inhibition of the endocytosis routing effect is low.

As expected for the lipid raft‐mediated/caveolar pathway, the progression of the internalized caveolae to early endosomes and later to lysosomes was absent or very slow;^11^, ^15^ therefore, no colocalization with lysosomes was observed, sparing the cargo from early degradation.^36^ This may have promoted partial escape of the cargo from the caveosomes, which could be observed in our experiments as diffuse intracellular fluorescence. This feature opens a path to further development of the endocytosis routing sequence presented here. Tagging the protein cargo with the large fluorescent protein r‐phycoerythrin allowed us to test the functionality of the intracellular protein. The selected mechanism left the protein cargo intact, as shown by its fluorescence even after hours of incubation. Moreover, the molecular recognition between the primary and secondary antibodies and between the primary antibody and its externally added antigen was functional, indicating the absence of degradation of the carrier–cargo complex.

Many techniques for intracellular delivery have emerged, including electroporation; microinjection as a physical method; and the use of cationic lipid constructs, protein transduction agents, nanobodies, and cell‐penetrating peptides,^22^, ^37^, ^38^, ^39^ including cyclic^40^, ^41^ and stapled^42^ derivatives. Despite the continued development of methods for the intracellular delivery of macromolecules, delivery of macromolecular cargoes is rare; furthermore, only 4% of successfully delivered molecules are proteins, and very few antibodies have been delivered.^43^ Although some carriers have been shown to translocate membranes with their macromolecular cargo, micromolar concentrations of such carriers and their cargo are required, precluding their therapeutic application,^44^ especially when toxicity also enters the picture.^32^

## 4 Conclusion

Our GM1 receptor‐based modular approach is a useful alternative to the currently available carriers because the very short, easily applied, and nontoxic WYKYW‐tag facilitates the advantageous lipid raft‐mediated/caveolar endocytosis in a carrier‐triggered manner, and it works at therapeutically relevant concentrations for cells expressing GM1. It is increasingly important to develop methods for cell‐ and tissue‐specific targeting of cargoes.^45^ While efforts have been made to achieve specific targeting,^46^, ^47^ selectivity toward cancer cells is still of great interest. The GM1‐dependent endocytosis of WYKYW‐tagged cargo offers selectivity for cell types that overexpress GM1, a characteristic of many tumor cells. This cell type‐dependent effect is strongly supported by the results of our experiments with HeLa and Jurkat cells, which display different amounts of GM1 on the cell surface.

## 5 Experimental Section

*Peptide Synthesis and Purification*: Peptide amides were synthesized on TentaGel R RAM resin using (7‐azabenzotriazol‐1‐yl)tetramethyluronium hexafluorophosphate as a coupling agent (HATU). Coupling was performed at room temperature with three equivalents amino acid excess for 3 h. For the PEG‐based construct, Fmoc‐Ebes was coupled three times consecutively after the peptide sequence. The peptides were cleaved with TFA/water/d,l‐dithiothreitol/triisopropylsilane (90:5:2.5:2.5) and then precipitated in ice‐cold diethyl ether. The resin was washed with acetic acid and water and subsequently filtered and lyophilized. Peptides were purified by RP‐HPLC on a C18 column. The HPLC eluents were 1) 0.1% TFA in water and 2) 0.1% TFA in ACN. The purity of the peptides was confirmed by analytical RP‐HPLC and ESI‐MS measurements.

*Isothermal Titration Calorimetry*: ITC was performed in pH 7.2 phosphate buffer solution using a MicroCal VP‐ITC microcalorimeter. In individual titrations, 15 µL of solution containing GM1:DPC 1:5 was injected into the ligand solution in the cell (the GM1:DPC mixture was prepared in the same buffer as the ligand in the cell) from a computer‐controlled 300 µL microsyringe at intervals of 300 s. All measurements were conducted at 35 °C. The concentration of the ligand in the cell was 15 µm, and the concentration of GM1 in the syringe was 300 µm. Control experiments were performed by injecting GM1 into a cell containing buffer but no ligand. The experiments were repeated twice. We occasionally observed tailing heat response curves both in the actual titrations and in background measurements. To test the effects of the tailing on the fitted parameters, we repeated specific titrations with 600 s time delay between injections (Figure S1c, Supporting Information). Fitted affinity parameters did not exhibit marked change with the experimental setup, indicating that the tailing is not related to the binding phenomenon. After background subtraction and spline baseline correction, the experimental data were fitted to one‐binding‐site or two‐independent‐site models (adjustable parameters: Δ*H*_b1_, *K*_d1_, *n*_1_, and Δ*H*_b2_, *K*_d2_, *n*_2_) using a generalized reduced gradient nonlinear least‐squares procedure. Residual heat of mixing was observed in the enthalpograms due to the inert counter ions and residual solvent in the peptide, protein, and lipid samples, which was corrected by including a constant correction term as an additional fitted parameter. Errors were calculated by jackknife resampling.

*NMR Experiments*: The ^1^H and STD NMR spectra were recorded on a Bruker AVANCE III 600 MHz spectrometer equipped with a 5 mm CP‐TCI triple‐resonance cryoprobe at 308 K. The compounds were dissolved in *d*_18_‐HEPES buffer (20 mm, pH 6.5) containing 10% D_2_O. ^1^H STD spectra were acquired with water suppression using excitation sculpting with pulsed gradients. For the ^1^H and STD measurements, the ligands and the GM1 concentrations were 20 µm. The samples were placed in 2.5 mm capillary NMR tubes. As a reference, STD experiments were also performed without the target in samples that contained the ligand species alone. STD NMR spectra were acquired using a series of 40 equally spaced 50 ms Gaussian‐shaped pulses for selective saturation of the protein, with a total saturation time of 2 s and a 50 ms spinlock to suppress the signal from the bicelles. The frequency of the on‐resonance saturation was set at 1.2 ppm, and the off‐resonance saturation frequency was set at 40.0 ppm. A total of 8k scans were collected for each pseudo‐2D experiment.

*Cell Culture*: HeLa cells were cultured in advanced MEM (Gibco, Life Technologies) supplemented with 10% fetal bovine serum (FBS, PAN‐Biotech). JN_2_B_4_D Jurkat cells were cultured in RPMI‐1640 medium (Gibco) supplemented with 5% FBS. Both media contained penicillin‐streptomycin (100 U mL^−1^, Gibco) and 2 mm L‐glutamine (Gibco). The cells were grown in a humidified incubator containing 5% CO_2_ at 37 °C.

*Preparation of Carrier–Protein Complexes*: To prepare peptide–NA complexes, biotinylated peptides were incubated with FITC‐NA in cell culture medium at a molar ratio of 4:1. The resulting solution containing the complexes was added to the cells at various concentrations. To prepare the antibody complexes, a solution of the biotinylated peptide was mixed with biotinylated monoclonal mouse anti‐human galectin‐1 (2c1/6) antibody^48^ and labeled or unlabeled NA at a molar ratio of 3:1:1; secondary r‐phycoerythrin‐conjugated goat anti‐mouse IgG (F‐[ab′]2, DakoCytomation) antibody or in some cases secondary Alexa Fluor 647‐conjugated F(ab′)2‐goat anti‐mouse IgG (Invitrogen) was then added to the solution at a 1:1 molar ratio with the primary antibody. HeLa cells were incubated with various concentrations of this complex.

*Flow Cytometry*: The internalization of peptides and peptide–NA complexes was determined by flow cytometric analysis. Cells (6 × 10^4^ in 24‐well plates) were grown at 37 °C for 24 h. After removal of the medium, the cells were washed with PBS and incubated with peptides or peptide complexes in MEM+1% FBS at 37 °C. The cells were then washed with PBS, harvested from the plates with 0.05% trypsin‐EDTA, and washed with PBS. Trypan blue (Reanal) and propidium iodide (Fluka) were added to the cells at final concentrations of 0.1% and 15 µm, respectively, in PBS immediately before the cells were subjected to flow cytometric analysis (FACSCalibur flow cytometer, BD Biosciences). The data were evaluated using FlowJo software (FlowJo, LLC). The fluorescence intensity of control cells and of the FITC‐NA was subtracted from the fluorescence intensity of the peptides and peptide–NA complexes. For the measurement of ganglioside GM1 content, HeLa and Jurkat cells were incubated with 8 µm FITC‐cholera toxin B subunit on ice for 30 min and then subjected to flow cytometric analysis as described above. For the in vitro competition assay, Jurkat cells were treated with 1 µm peptide alone or with 1, 5, or 10 µm galectin‐1. For the endocytosis inhibition experiments, HeLa cells were preincubated at 37 °C with 5 mg mL^−1^ methyl‐β‐cyclodextrin (MBCD) for 60 min, 10 µm wortmannin for 60 min, or 10 µg mL^−1^ chlorpromazine for 30 min. The cells were then incubated with 1 µm peptide–NA complexes at 37 °C for 60 min, treated with trypan blue, and subjected to flow cytometric analysis as described above. All experiments were performed in triplicate.

*Live Confocal Laser Scanning Microscopy*: HeLa cells were plated overnight culturing in MEM+10% FBS at 1.25 × 10^4^ cells per cm^2^ (or 1.5 × 10^4^ cells per channel) on six‐chamber µ‐Slides VI 0.4 (ibidi). The cells were washed with PBS and incubated with the studied complexes in MEM+1% FBS medium at different concentrations for different incubation times at 37 °C. The cells then were washed with PBS; when antibody‐complexes were used, they were also washed with 100 mm β‐lactose (Sigma‐Aldrich) and the biotinylated peptide–NA complex without the primary and secondary antibody to remove surface–bound complexes. The cells were stained with 100 ng mL^−1^ Hoechst 33342 (Sigma‐Aldrich) in MEM medium for 30 min at 37 °C. In some experiments, after Hoechst staining, the cells were labeled with LysoTracker Red (Life Technologies) at 75 nm for 30 min at 37 °C according to the manufacturer's instructions. For the cholera toxin colocalization experiments, cells were coincubated with 5 µm FITC‐labeled CTX‐B subunit (Sigma‐Aldrich). For the structural test of the antibody complex, cells were treated with the complex for 3 h at 500 nm, then the cells were fixed with 1.6% paraformaldehyde for 15 min at room temperature, permeabilized with 0.01% saponin for 10 min, and then cells were stained with 350 nm Atto 488‐conjugated galectin‐1 for 30 min at room temperature.

For the IgG complex measurements, cell membranes were visualized after a 5 min treatment with FITC‐labeled WGA lectin at 0.2 µg mL^−1^ at room temperature after incubation with the complex. The cells were incubated in Leibovitz's L‐15 medium (Life Technologies) during microscopic analysis. FITC–NA complexes were treated with 0.1% Trypan blue to quench extracellular fluorescence. To observe the localization of the cargo, cell fluorescence was analyzed using a Leica SP5 AOBS confocal laser scanning microscope using the 405 nm UV diode (for Hoechst staining), the 488 nm argon laser line (for FITC and Atto 488 staining), the 543 nm HeNe laser line (for r‐phycoerythrin and LysoTracker Red staining), and the 633 HeNe laser line (for Alexa Fluor 647 staining). For emission detection, an appropriate spectral filter was used for each channel.

*Image Analysis*: To identify cells and extract their properties, we used Mask R‐CNN, a deep learning‐based image segmentation platform,^49^ and CellProfiler software^50^ for feature extraction. First, cell nuclei were identified based on the Hoechst signal using a very heavily augmented training set of The Data Science Bowl 2018 competition. The augmentation was performed by learning image styles and generating synthetic images of similar types with Pix2Pix, a generative adversarial network (GAN) deep network.^51^ A Mask R‐CNN network was then trained, and individual nuclei were inferred. The cytoplasm was approximated with a watershed region propagation algorithm on the FITC‐WGA lectin channel. Using the detected objects (nucleus and cytoplasm) as masks, cellular features such as r‐phycoerythrin intensity values, textural properties, and morphological descriptors were extracted. For the final statistical analysis, the integrated intensities of individual cells were used.

*Cytotoxicity Assay*: The kinetics of cell reaction to peptide treatment was monitored by impedance measurement at 10 kHz (RTCA SP instrument, ACEA Biosciences, San Diego, CA). Impedance measurement is noninvasive, label‐free, and real‐time and correlates linearly with the adherence, growth, number, and viability of the cells. For background measurements, 50 µL of cell culture medium was added to the wells, and HeLa cells were seeded at a density of 6 × 10^3^ cells per well on noncoated 96‐well plates with integrated gold electrodes (E‐plate 96, ACEA Biosciences). At the beginning of the plateau phase of growth, the cells were treated with peptide solutions at 0.1, 0.5, 1, 5, and 10 µm, and the effects of treatment were followed for 24 h. Triton X‐100 detergent (1 mg mL^−1^) was used as a reference compound to induce cell death. The cell index was defined as Rn‐Rb at each time point of measurement, where Rn is the cell‐electrode impedance of the well when it contains cells and Rb is the background impedance of the well containing medium alone.

*Statistical Analysis*: Statistical analysis included one‐way analysis of variance (ANOVA) with post hoc Tukey honestly significant difference test (*p < 0.1; **p < 0.01; ***p < 0.001; ****p < 0.0001) and unpaired Student's *t*‐test (*p < 0.05; **p < 0.01; ***p < 0.001; ****p < 0.0001).

## Conflict of Interest

The authors declare that a patent application has been submitted covering the findings presented in this manuscript.

## Supporting information

Supporting Information

## References

[1] K. Fosgerau , T. Hoffmann , Drug Discov. Today 2015, 20, 122. 25450771 10.1016/j.drudis.2014.10.003

[2] M. Sánchez‐Navarro , M. Teixidó , E. Giralt , Nat. Chem. 2017, 9, 727. 28754938 10.1038/nchem.2837

[3] L. Pelkmans , T. Burli , M. Zerial , A. Helenius , Cell 2004, 118, 767. 15369675 10.1016/j.cell.2004.09.003

[4] L. Pelkmans , J. Kartenbeck , A. Helenius , Nat. Cell Biol. 2001, 3, 473. 11331875 10.1038/35074539

[5] A. E. Smith , A. Helenius , Science 2004, 304, 237. 15073366 10.1126/science.1094823

[6] V. Pietiäinen , V. Marjomäki , P. Upla , L. Pelkmans , A. Helenius , T. Hyypiä , Mol. Biol. Cell 2004, 15, 4911. 15356270 10.1091/mbc.E04-01-0070 PMC524743

[7] R. Montesano , J. Roth , A. Robert , L. Orci , Nature 1982, 296, 651. 7070509 10.1038/296651a0

[8] R. Fajka‐Boja , A. Blasko , F. Kovacs‐Solyom , G. Szebeni , G. Toth , E. Monostori , Cell. Mol. Life Sci. 2008, 65, 2586. 18581052 10.1007/s00018-008-8143-x PMC11131775

[9] L. Pelkmans , A. Helenius , Traffic 2002, 3, 311. 11967125 10.1034/j.1600-0854.2002.30501.x

[10] S. Mayor , K. G. Rothberg , F. R. Maxfield , Science 1994, 264, 1948. 7516582 10.1126/science.7516582

[11] A. L. Kiss , E. Botos , J. Cell. Mol. Med. 2009, 13, 1228. 19382909 10.1111/j.1582-4934.2009.00754.x PMC4496137

[12] R. R. Sprenger , R. D. Fontijn , J. Van Marle , H. Pannekoek , A. J. Horrevoets , Biochem. J. 2006, 400, 401. 16886909 10.1042/BJ20060355 PMC1698592

[13] P. Moscariello , D. Y. W. Ng , M. Jansen , T. Weil , H. J. Luhmann , J. Hedrich , Adv. Sci. 2018, 5, 1700897. 10.1002/advs.201700897 PMC5979778 29876217

[14] M. Zorko , Ü. Langel , Adv. Drug Deliv. Rev. 2005, 57, 529. 15722162 10.1016/j.addr.2004.10.010

[15] T. Matsubara , R. Otani , M. Yamashita , H. Maeno , H. Nodono , T. Sato , Biomacromolecules 2017, 18, 355. 28051846 10.1021/acs.biomac.6b01262

[16] D. O'Sullivan , Comput. Ind. 2002, 47, 77.

[17] M. M. Fuster , J. D. Esko , Nat. Rev. Cancer 2005, 5, 526. 16069816 10.1038/nrc1649

[18] U. Krengel , P. A. Bousquet , Front. Immunol. 2014, 5, 325. 25101077 10.3389/fimmu.2014.00325 PMC4104838

[19] S. K. Fischer , J. Yang , B. Anand , K. Cowan , R. Hendricks , J. Li , G. Nakamura , A. Song , mAbs 2012, 4, 623. 22820463 10.4161/mabs.20814 PMC3499303

[20] J. H. Oh , S.‐E. Chong , S. Nam , S. Hyun , S. Choi , H. Gye , S. Jang , J. Jang , S. W. Hwang , J. Yu , Y. Lee , Adv. Sci. 2018, 5, 1800240. 10.1002/advs.201800240 PMC6096998 30128238

[21] B. R. McNaughton , J. J. Cronican , D. B. Thompson , D. R. Liu , Proc. Natl. Acad. Sci. USA 2009, 106, 6111. 19307578 10.1073/pnas.0807883106 PMC2659711

[22] J. A. Zuris , D. B. Thompson , Y. Shu , J. P. Guilinger , J. L. Bessen , J. H. Hu , M. L. Maeder , J. K. Joung , Z.‐Y. Chen , D. R. Liu , Nat. Biotechnol. 2015, 33, 73. 25357182 10.1038/nbt.3081 PMC4289409

[23] W. B. Kauffman , S. Guha , W. C. Wimley , Nat. Commun. 2018, 9, 2568. 29967329 10.1038/s41467-018-04874-6 PMC6028423

[24] M. C. Morris , J. Depollier , J. Mery , F. Heitz , G. Divita , Nat. Biotechnol. 2001, 19, 1173. 11731788 10.1038/nbt1201-1173

[25] S. André , C. E. P. Maljaars , K. M. Halkes , H.‐J. Gabius , J. P. Kamerling , Bioorg. Med. Chem. Lett. 2007, 17, 793. 17095217 10.1016/j.bmcl.2006.10.067

[26] C. E. P. Maljaars , S. André , K. M. Halkes , H.‐J. Gabius , J. P. Kamerling , Anal. Biochem. 2008, 378, 190. 18471425 10.1016/j.ab.2008.04.023

[27] S. Andre , C. J. Arnusch , I. Kuwabara , R. Russwurm , H. Kaltner , H.‐J. Gabius , R. J. Pieters , Bioorg. Med. Chem. 2005, 13, 563. 15598577 10.1016/j.bmc.2004.09.053

[28] E. Wéber , A. Hetényi , B. Váczi , É. Szolnoki , R. Fajka‐Boja , V. Tubak , É. Monostori , T. A. Martinek , ChemBioChem 2010, 11, 228. 19938027 10.1002/cbic.200900502

[29] J. Kopitz , C. von Reitzenstein , M. Burchert , M. Cantz , H.‐J. Gabius , J. Biol. Chem. 1998, 273, 11205. 9556610 10.1074/jbc.273.18.11205

[30] L. Cantù , M. Corti , E. Del Favero , A. Raudino , J. Phys.: Condens. Matter 2000, 12, A321.

[31] R. G. Parton , J. Histochem. Cytochem. 1994, 42, 155. 8288861 10.1177/42.2.8288861

[32] S. El‐Andaloussi , P. Jarver , H. J. Johansson , U. Langel , Biochem. J. 2007, 407, 285. 17627607 10.1042/BJ20070507 PMC2049024

[33] C. F. Brewer , Glycoconjugate J. 2002, 19, 459. 10.1023/B:GLYC.0000014075.62724.d0 14758069

[34] Z. Qian , T. Liu , Y.‐Y. Liu , R. Briesewitz , A. M. Barrios , S. M. Jhiang , D. Pei , ACS Chem. Biol. 2012, 8, 423. 23130658 10.1021/cb3005275 PMC3574231

[35] J. He , L. G. Baum , J. Biol. Chem. 2004, 279, 4705. 14617626 10.1074/jbc.M311183200

[36] V. P. Torchilin , Annu. Rev. Biomed. Eng. 2006, 8, 343. 16834560 10.1146/annurev.bioeng.8.061505.095735

[37] A. Erazo‐Oliveras , K. Najjar , L. Dayani , T.‐Y. Wang , G. A. Johnson , J.‐P. Pellois , Nat. Methods 2014, 11, 861. 24930129 10.1038/nmeth.2998 PMC4131206

[38] M. Akishiba , T. Takeuchi , Y. Kawaguchi , K. Sakamoto , H.‐H. Yu , I. Nakase , T. Takatani‐Nakase , F. Madani , A. Gräslund , S. Futaki , Nat. Chem. 2017, 9, 751. 28754944 10.1038/nchem.2779

[39] H. D. Herce , D. Schumacher , A. F. Schneider , A. K. Ludwig , F. A. Mann , M. Fillies , M.‐A. Kasper , S. Reinke , E. Krause , H. Leonhardt , Nat. Chem. 2017, 9, 762. 28754949 10.1038/nchem.2811

[40] G. Lättig‐Tünnemann , M. Prinz , D. Hoffmann , J. Behlke , C. Palm‐Apergi , I. Morano , H. D. Herce , M. C. Cardoso , Nat. Commun. 2011, 2, 453. 21878907 10.1038/ncomms1459 PMC3265364

[41] Z. Qian , J. R. LaRochelle , B. Jiang , W. Lian , R. L. Hard , N. G. Selner , R. Luechapanichkul , A. M. Barrios , D. Pei , Biochemistry 2014, 53, 4034. 24896852 10.1021/bi5004102 PMC4075989

[42] L. D. Walensky , A. L. Kung , I. Escher , T. J. Malia , S. Barbuto , R. D. Wright , G. Wagner , G. L. Verdine , S. J. Korsmeyer , Science 2004, 305, 1466. 15353804 10.1126/science.1099191 PMC1360987

[43] A. Gautam , H. Singh , A. Tyagi , K. Chaudhary , R. Kumar , P. Kapoor , G. P. S. Raghava , Database 2012, 2012, bas015. 22403286 10.1093/database/bas015 PMC3296953

[44] K. Saar , M. Lindgren , M. Hansen , E. Eiríksdóttir , Y. Jiang , K. Rosenthal‐Aizman , M. Sassian , Ü. Langel , Anal. Biochem. 2005, 345, 55. 16137634 10.1016/j.ab.2005.07.033

[45] G. Guidotti , L. Brambilla , D. Rossi , Trends Pharmacol. Sci. 2017, 38, 406. 28209404 10.1016/j.tips.2017.01.003

[46] K. Bremner , L. Seymour , C. Pouton , Curr. Opin. Mol. Ther. 2001, 3, 170. 11338930

[47] S. Dissanayake , W. A. Denny , S. Gamage , V. Sarojini , J. Controlled Release 2017, 250, 62. 10.1016/j.jconrel.2017.02.006 28167286

[48] G. J. Szebeni , É. Kriston‐Pál , P. Blazsó , R. L. Katona , J. Novák , E. Szabó , Á. Czibula , R. Fajka‐Boja , B. Hegyi , F. Uher , PLoS One 2012, 7, e41372. 22844466 10.1371/journal.pone.0041372 PMC3402504

[49] K. He , G. Gkioxari , P. Dollár , R. Girshick , Mask R‐CNN, 2017, arXiv: 1703.06870. 10.1109/TPAMI.2018.2844175 29994331

[50] A. E. Carpenter , T. R. Jones , M. R. Lamprecht , C. Clarke , I. H. Kang , O. Friman , D. A. Guertin , J. H. Chang , R. A. Lindquist , J. Moffat , Genome Biol. 2006, 7, R100. 17076895 10.1186/gb-2006-7-10-r100 PMC1794559

[51] P. Isola , J‐Y. Zhu , T. Zhou , A. A. Efros , Image‐to‐Image Translation with Conditional Adversarial Networks, CoRR, 2016, abs/1611.07004.

