# Supplementary material for: Routing Nanomolar Protein Cargoes to Lipid Raft‐Mediated/Caveolar Endocytosis through a Ganglioside GM1‐Specific Recognition Tag
Source: Adv Sci (Weinh). 2020 Jan 9;7(4):1902621. doi: 10.1002/advs.201902621 (PMC7029632; doi:10.1002/advs.201902621)

Copyright WILEY-VCH Verlag GmbH & Co. KGaA, 69469 Weinheim, Germany, 2020.

Supporting Information

for *Adv. Sci.,* DOI: 10.1002/advs.201902621

Routing Nanomolar Protein Cargoes to Lipid Raft-Mediated/Caveolar Endocytosis through a Ganglioside GM1-Specific Recognition Tag

*Norbert Imre, Anasztázia Hetényi, Enik Szabó, Brigitta Bodnár, Abel Szkalisity, Ilona Gróf, Alexandra Bocsik, Mária A. Deli, Peter Horvath, Ágnes Czibula, Éva Monostori, and Tamás A. Martinek**

Supporting Information

Routing nanomolar protein cargoes to lipid raft-mediated/caveolar endocytosis through a ganglioside GM1-specific recognition tag

*Norbert Imre, Anasztázia Hetényi, Enikő Szabó, Brigitta Bodnár, Abel Szkalisity, Ilona Gróf, Alexandra Bocsik, Mária A. Deli, Peter Horvath, Ágnes Czibula, Éva Monostori & Tamás A. Martinek**

Contents

Figure S1. 2
Figure S2 2
Figure S3 3
Figure S4 4
Figure S5 4
Figure S6 4
Figure S7 5
Figure S8 6
Figure S9 6
Figure S10 7
Peptide characterization data 8
Supplementary Table 1 18
Full STD spectra 20
Raw ITC data for Figure 2 22
Raw ITC data for Figure S1 22
Raw ITC data for Figure S5 23

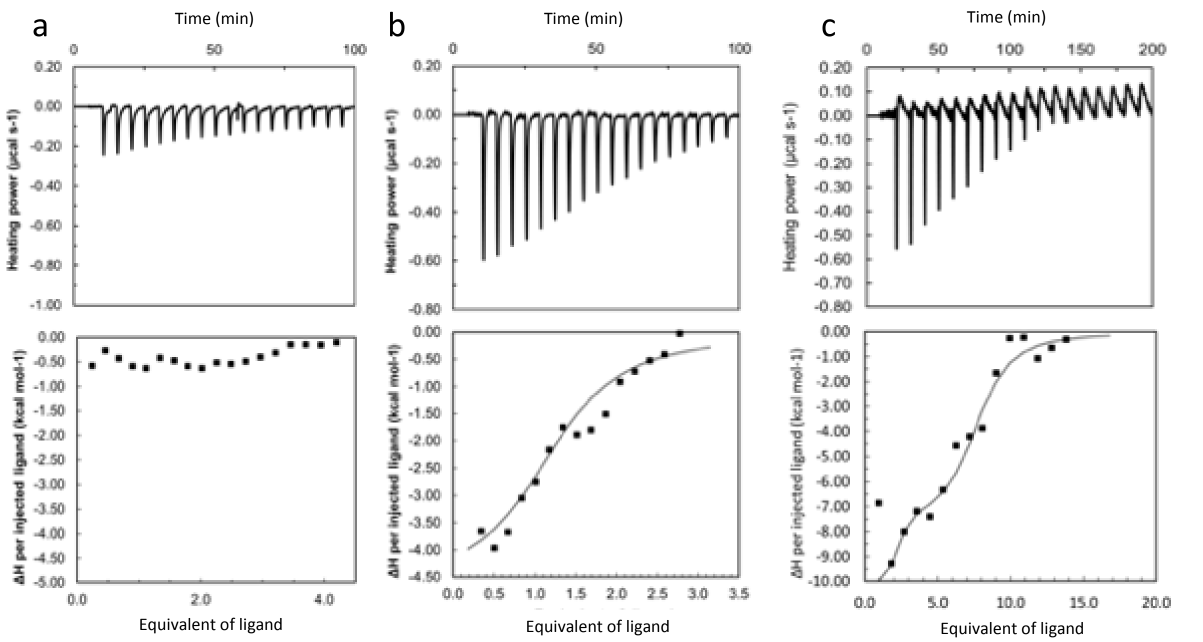

Figure S1. ITC enthalpogram of peptide WYKYW titrated with asialo-GM1. Titration was carried out with asialo-GM1:DPC 1:5 bicelles (**a**). ITC enthalpogram of peptide WYKYW titrated with GM3. Titration was carried out with GM3:DPC 1:5 bicelles (**b**).ITC enthalpogram of peptide NA(biotinyl-Penetratin-GG-WYKYW)$_4$ titrated with GM1. Titration was carried out with GM1:DPC 1:5 bicelles and an increased time delay of 600 s between injections. Fitted affinities did not show marked change with the altered experimental setup (n_1 = 1.8, K_{D1}= 15 nM, n_2 = 5.0 K_{D2}= 800 nM) (**c**)

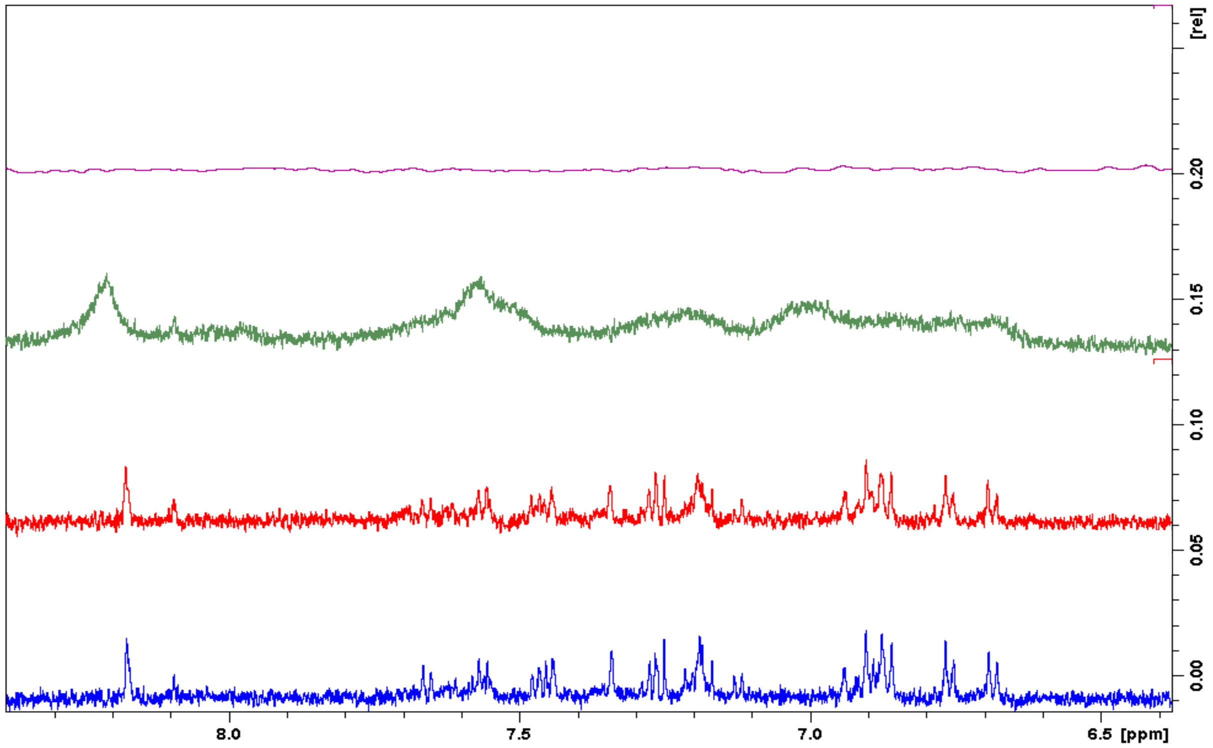

Figure S2. NMR detection of the WKYKW – GM1 interaction: ^{1}H STD of 20 µM WYKYW + 100 µM DPC + 20 µM ganglioside GM1 (purple), ^{1}H NMR of 20 µM WYKYW + 100 µM

DPC + 20 μM ganglioside GM1 (green), ^{1}H NMR of 20 μM WYKYW + 100 μM DPC (red), ^{1}H NMR of 20 μM WYKYW (blue).

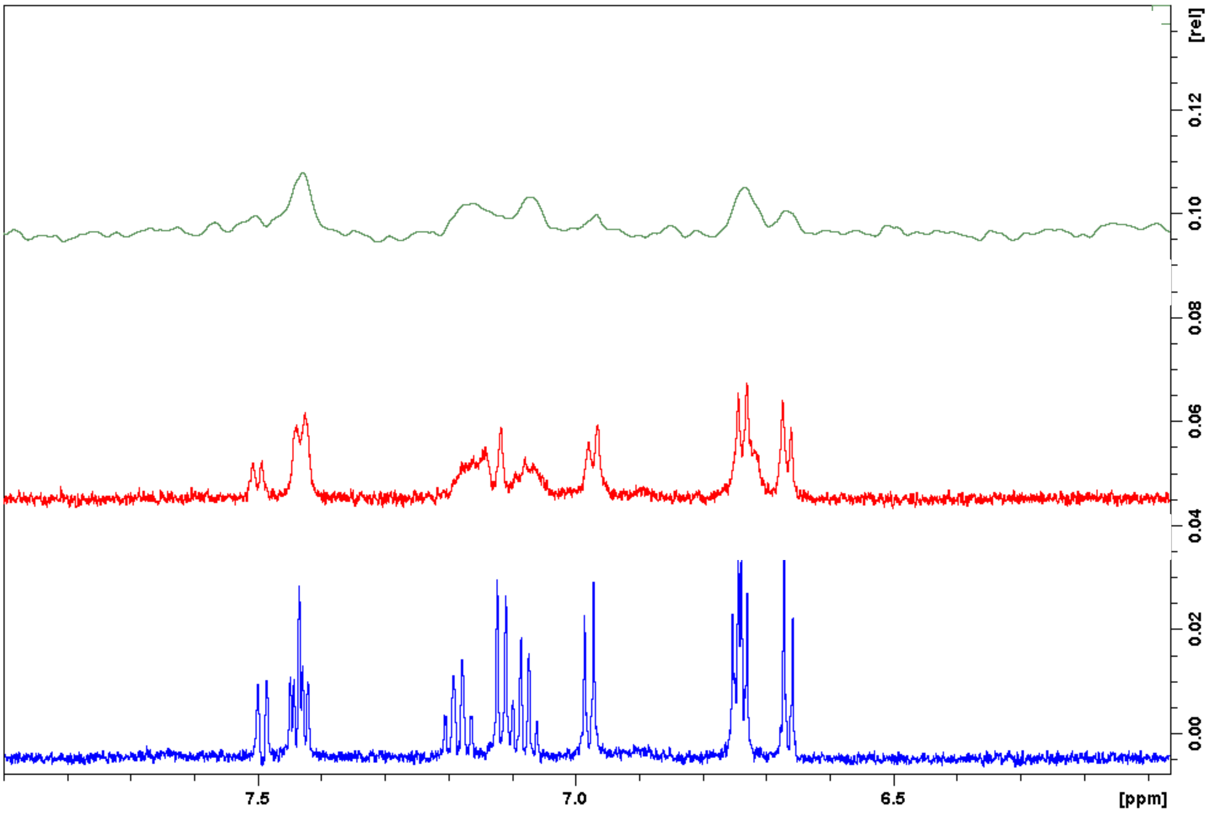

Figure S3. NMR detection of the WKYKW – GM3 interaction: ^{1}H STD of 20 μM WYKYW + 100 μM DPC + 20 μM ganglioside GM3 (green), ^{1}H NMR of 20 μM WYKYW + 100 μM DPC + 20 μM ganglioside GM3 (red), ^{1}H NMR of 20 μM WYKYW (blue).

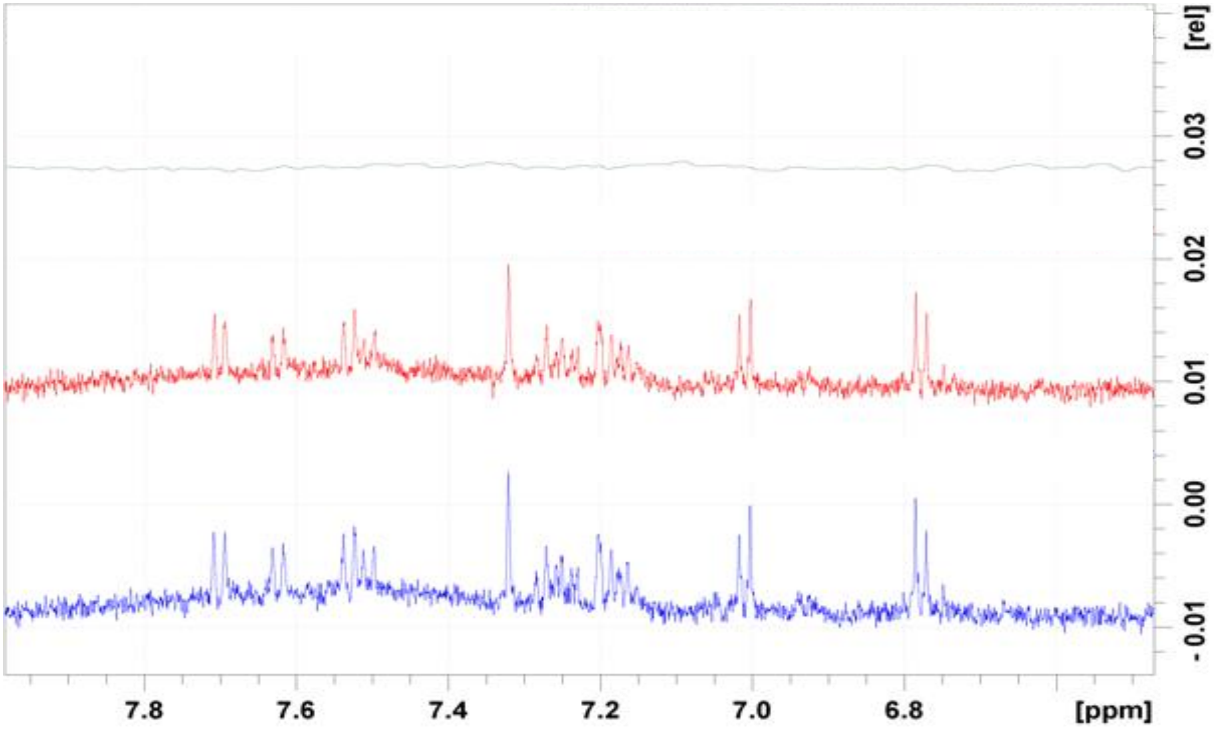

Figure S4. NMR detection of the WKYKW – asialo-GM1 interaction: ^{1}H STD of 20 µM WYKYW + 100 µM DPC + 20 µM asialo-GM1 (green), ^{1}H NMR of 20 µM WYKYW + 100 µM DPC + 20 µM asialo-GM1 (red), ^{1}H NMR of 20 µM WYKYW (blue).

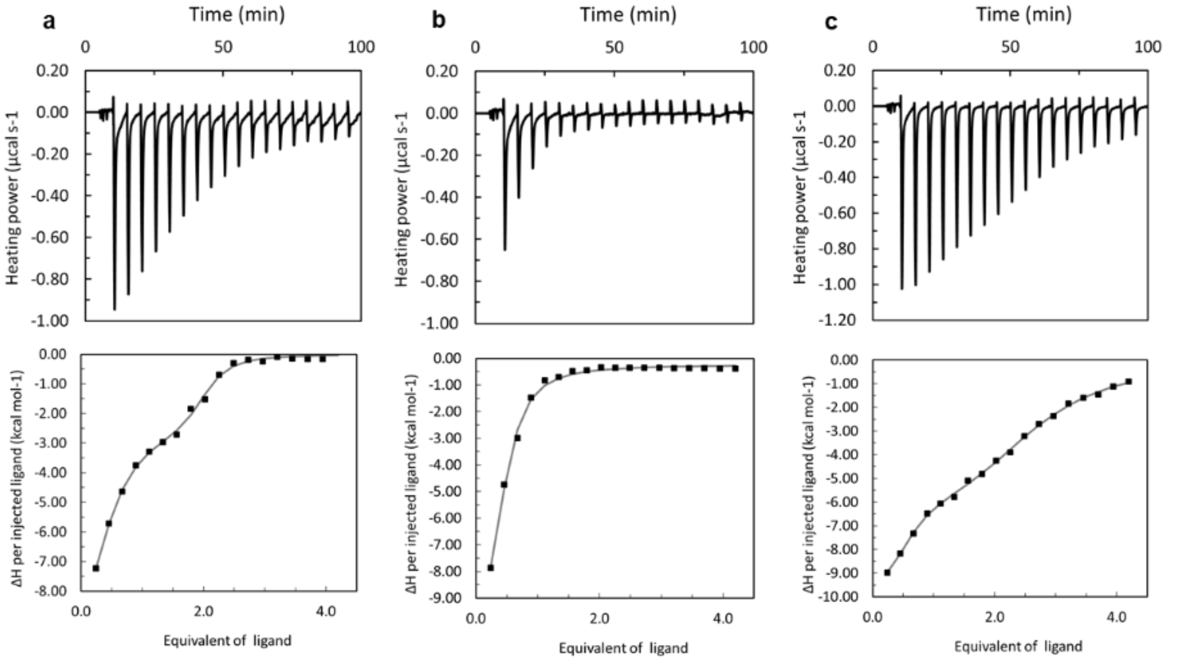

Figure S5. ITC detection of interactions of GM1 with CFU-tagged WYKYW derivatives. Enthalpograms of (**a**) peptide CFU-WYKYW (n_1 = 0.5, K_{D1} = 163.94 nM, n_2 = 1.5, K_{D2} = 1379.26 nM), (**b**) peptide CFU-WYKYW-GG-Penetratin (n=0.5, K_D= 894 nM) and (**c**) peptide CFU-Penetratin-GG-WYKYW (n_1 = 0.5, K_{D1} = 152 nM, n_2 = 1.78 K_{D2} = 3420 nM). Titrations were carried out with GM1:DPC 1:5 bicelles.

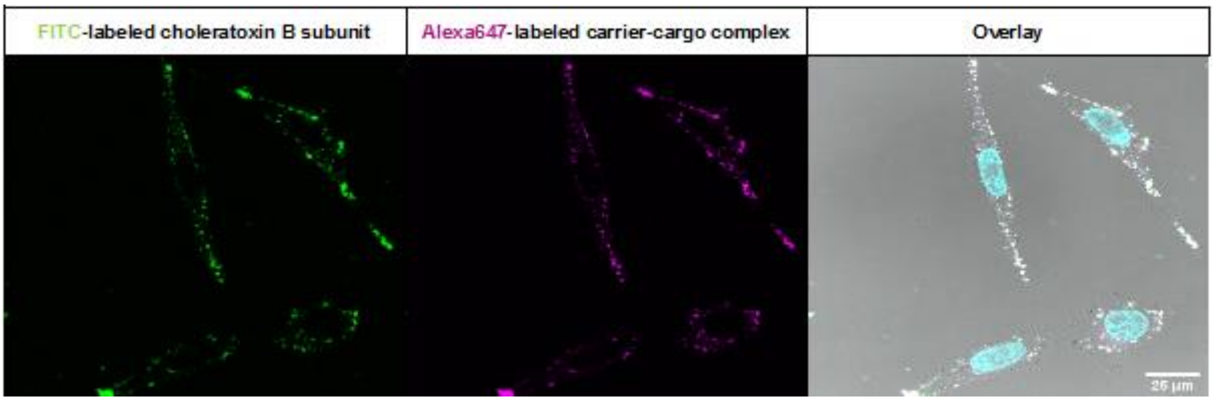

Figure S6. Co-localization after internalization of the carrier (NA(biotinyl-Penetratin-GG-WYKYW)$_4$) – cargo (primary and secondary IgGs) complex at 80 nM (indicated in magenta, secondary anibody tagged with Alexa Fluor 647) with the FITC-labeled cholera toxin B subunit at 5 µM (indicated in green) after 1 hour. Nuclei are stained with Hoechst33342 (indicated in cyan).

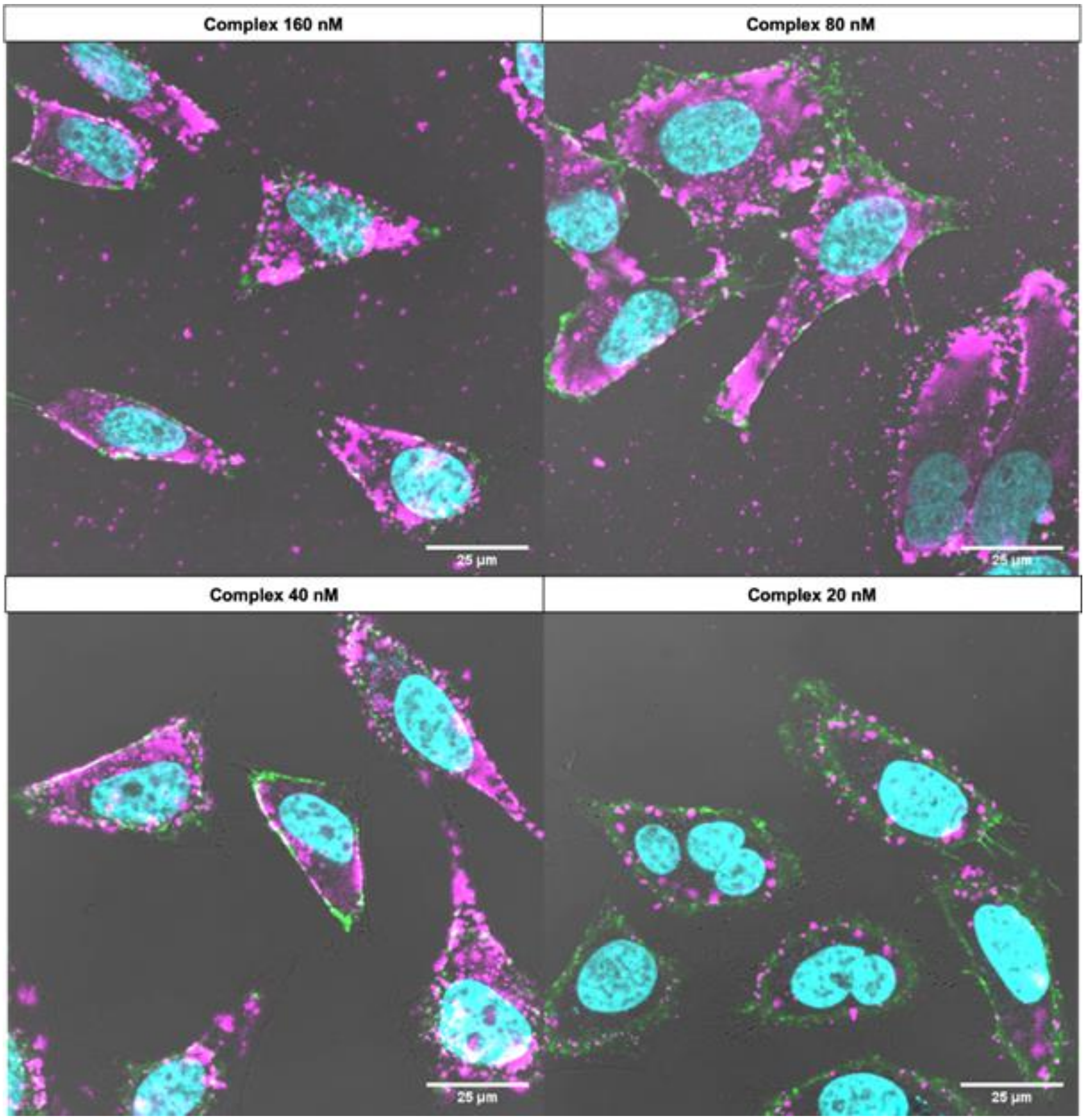

Figure S7. CLSM images of delivery of the IgG complex into HeLa cells at different concentrations after 3 hours. R-phycoerythrin-conjugated antibody is indicated in magenta, green staining defines cell membranes (WGA-FITC). Nuclei are stained with Hoechst33342 (indicated in cyan).

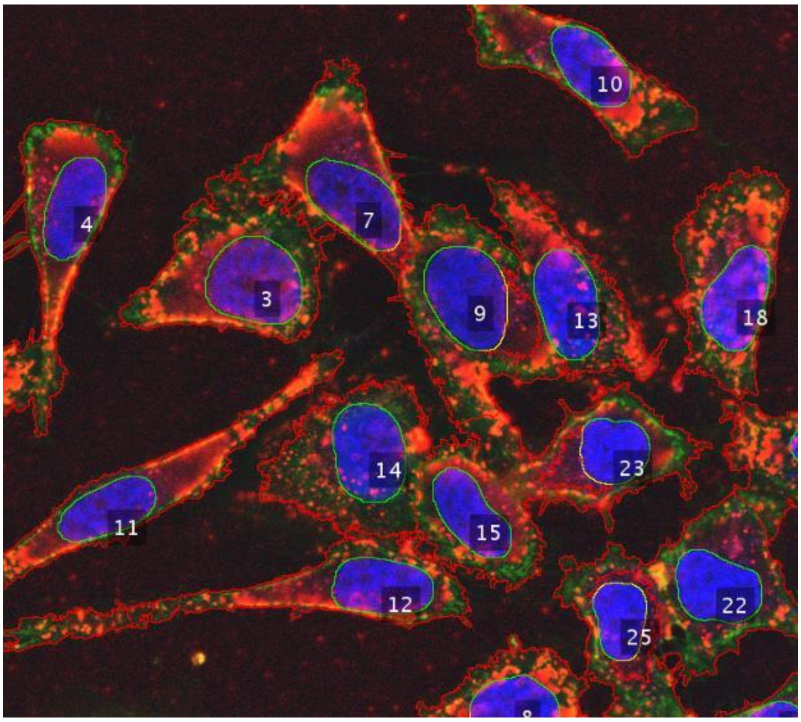

Figure S8. An example of the artificial intelligence-aided quantification of the CLSM images. HeLa cells were incubated with the IgG complex at 160 nM for 3 hours. R-phycoerythrin-conjugated antibody is indicated in red, membrane marker FITC-WGA in green. Hoechst labeled blue nuclei were identified with a deep learning-based platform, and cytoplasm was approximated with an algorithm. Red intensity values were extracted.

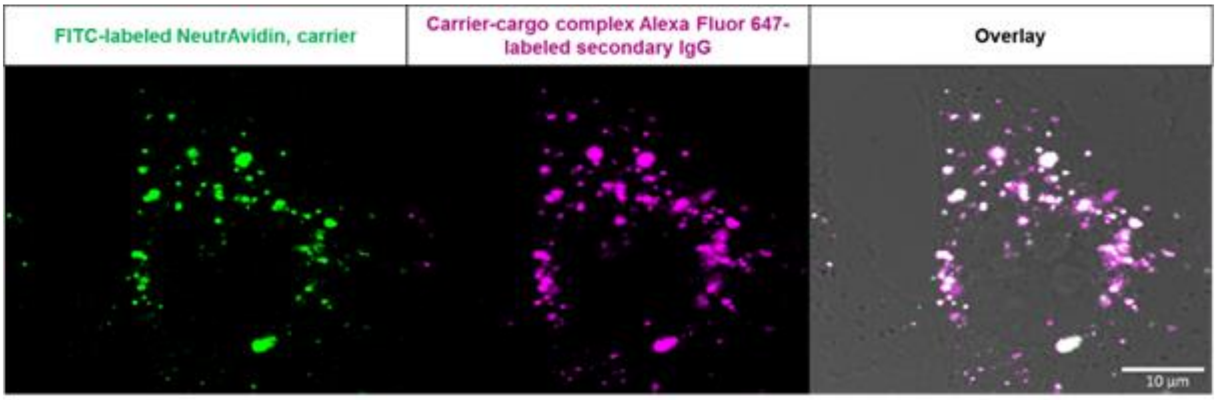

Figure S9. Internalization of the carrier-cargo complex into HeLa cells at 500 nM after 3 hours as determined by live confocal laser scanning microscopy. The FITC-labeled NeutrAvidin is indicated in green, the Alexa Fluor 647-labeled secondary IgG is indicated in magenta. Overlay of the images shows co-localization in the cell.

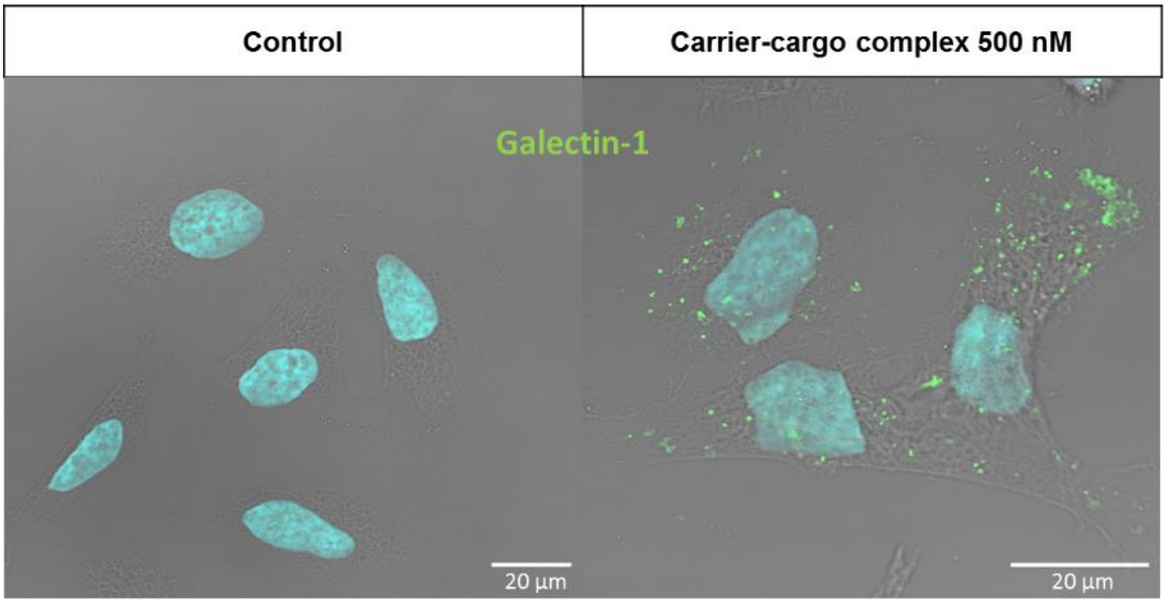

Figure S10. Internalization of the carrier-cargo complex biotinyl-Penetratin-GG-WYKYW:NeutrAvidin:Primary IgG 2c1/6 (anti-galectin-1) at 500 nM after 3 hours. After the incubation period, the cells were fixed, permeabilized and Atto488-labeled galectin-1 (350 nM, indicated in green) was applied to visualize the internalized carrier-cargo complex. Nuclei are stained with Hoechst33342 (indicated in cyan). Controls cells were incubated in culture media without the carrier-cargo complex, then were treated the same way with Atto488-labeled galectin-1.

Peptide characterization data

HPLC chromatograms and MS spectra for each sequence are shown below.

WYKYW (WYKYW-NH_2)

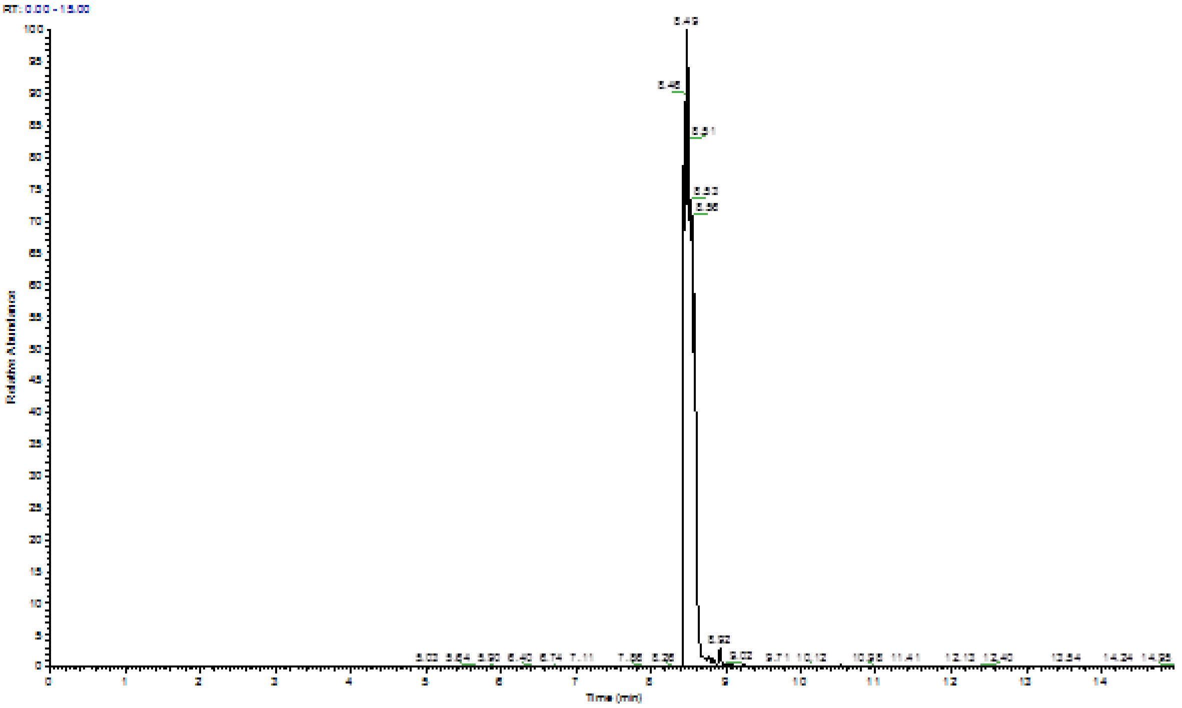

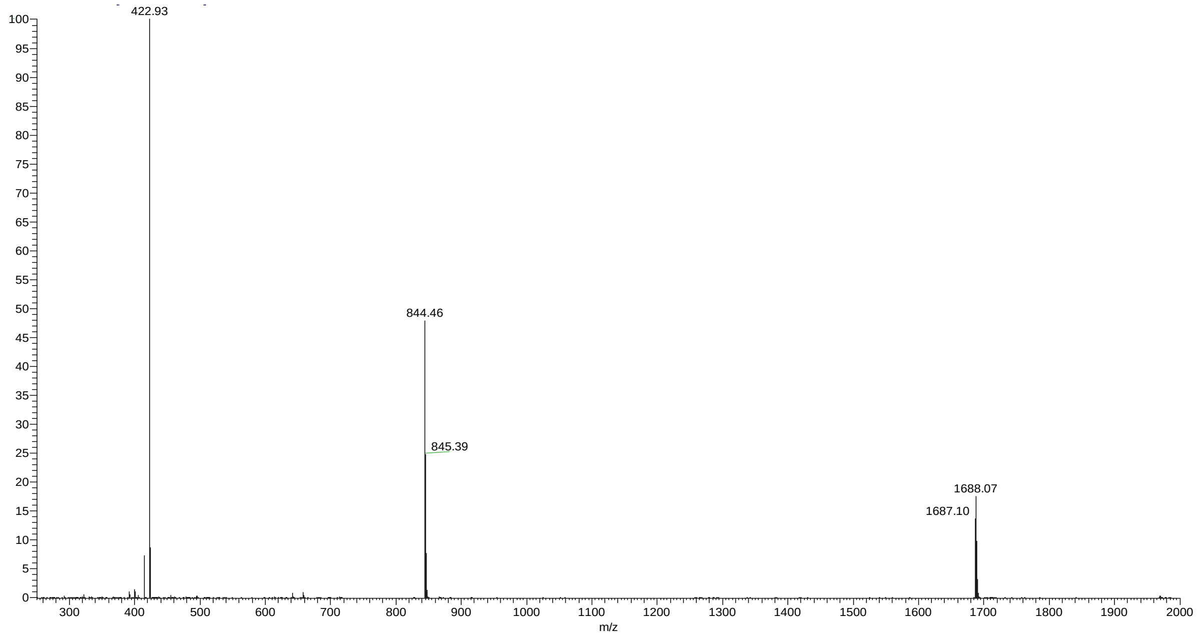

CFU-WYKYW (5(6)-FAM-WYKYW-NH_2)

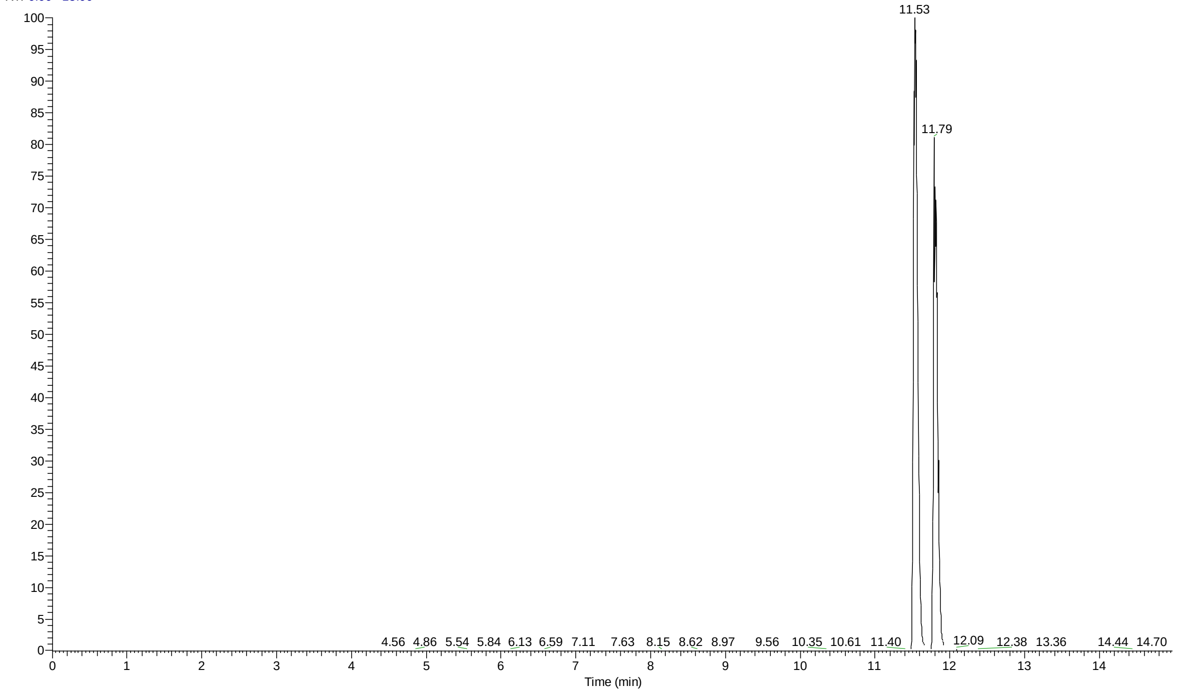

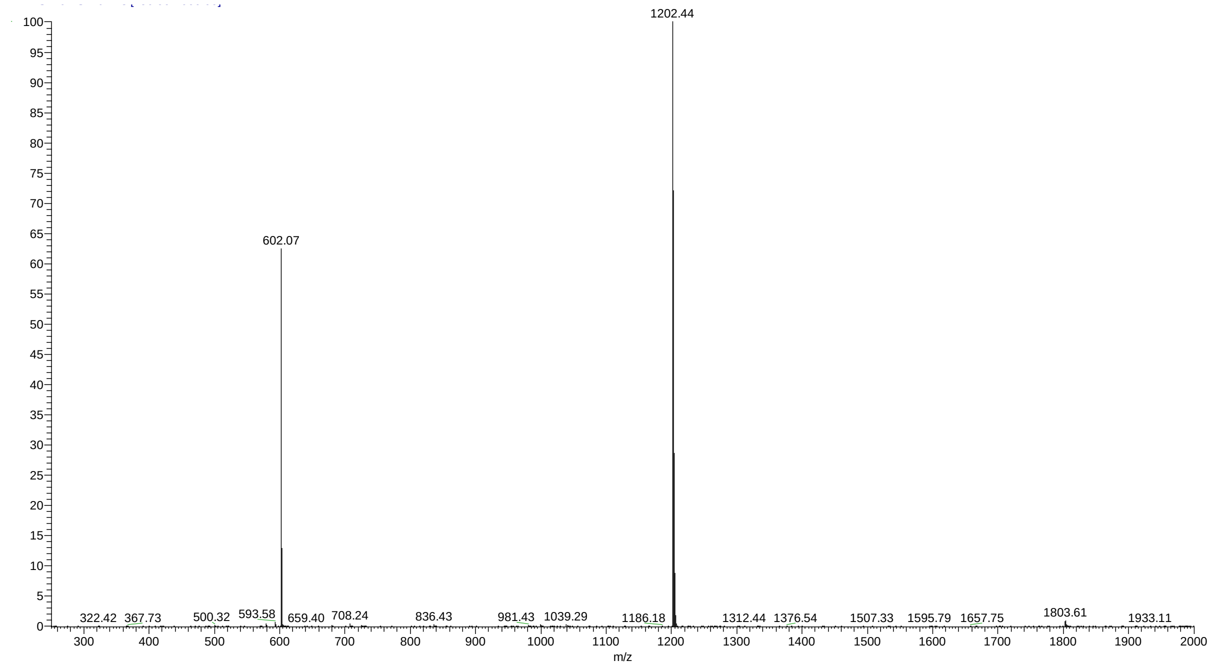

CFU-Penetratin (5(6)-FAM-RQIKIWFQNRRMKWKK-NH$_2$)

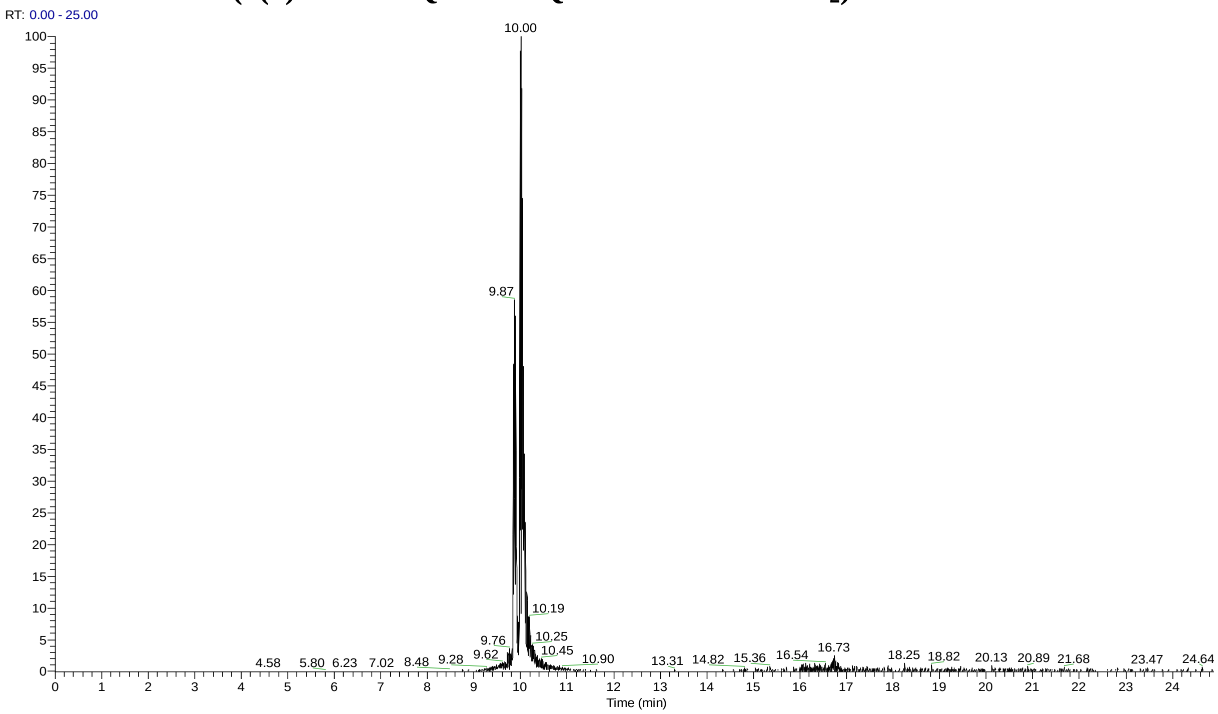

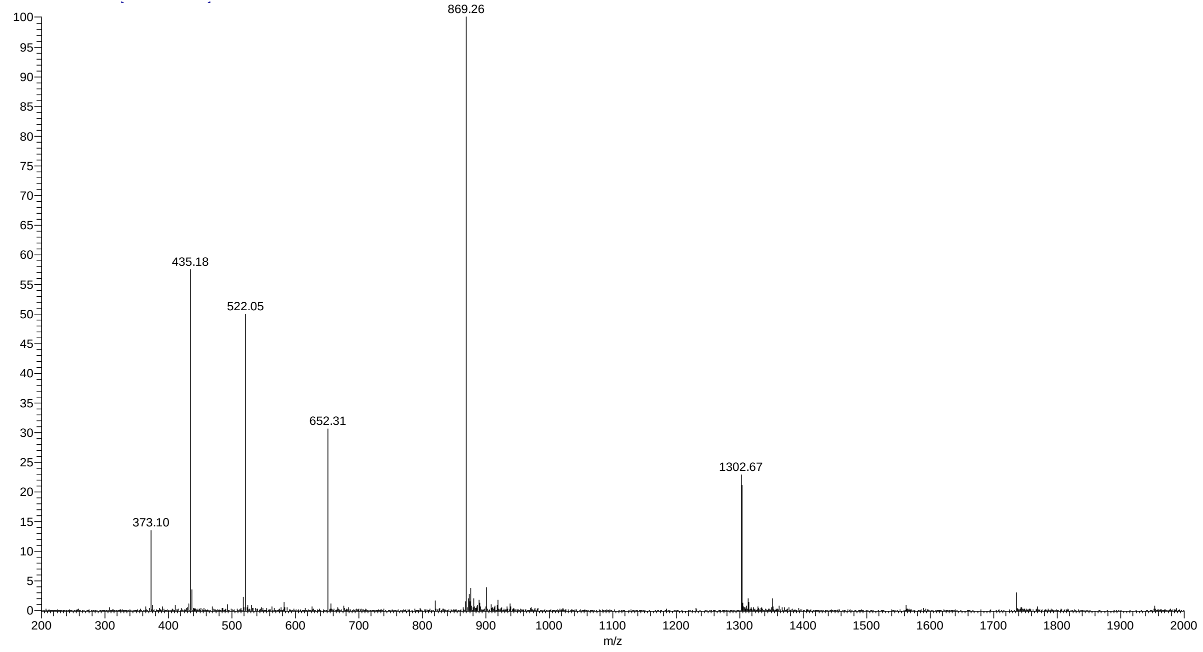

WYKYW-GG-Penetratin (WYKYWGGRQIKIWFQNRRMKWKK-NH_2)

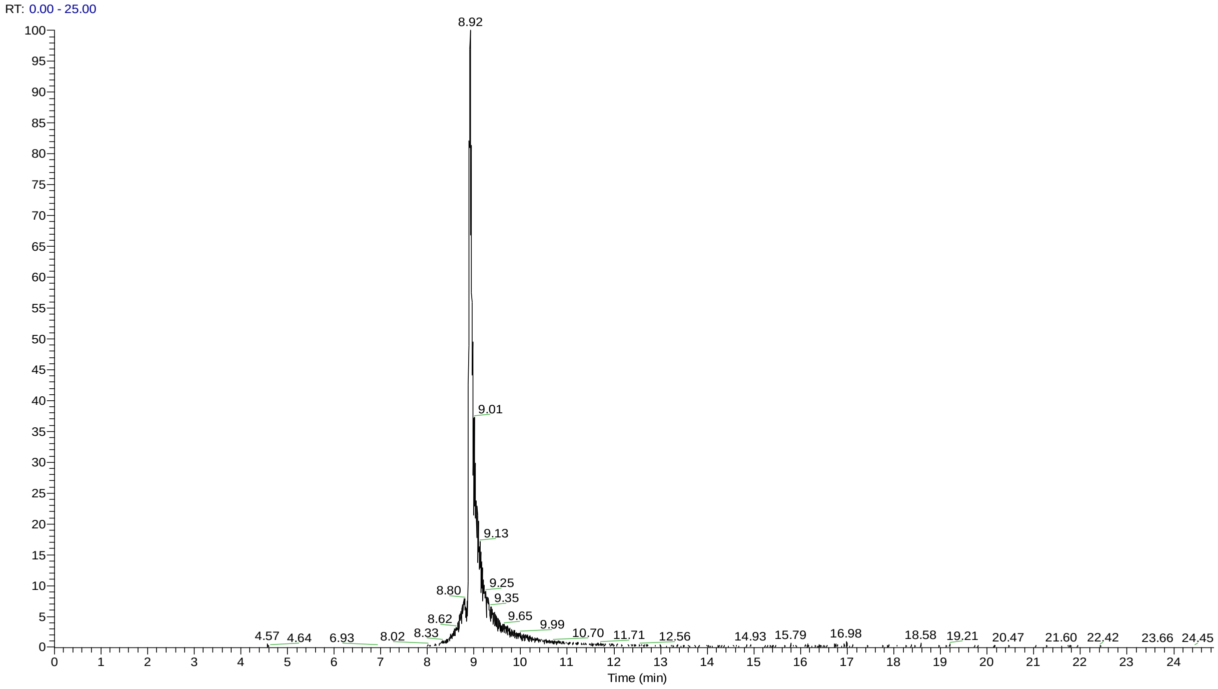

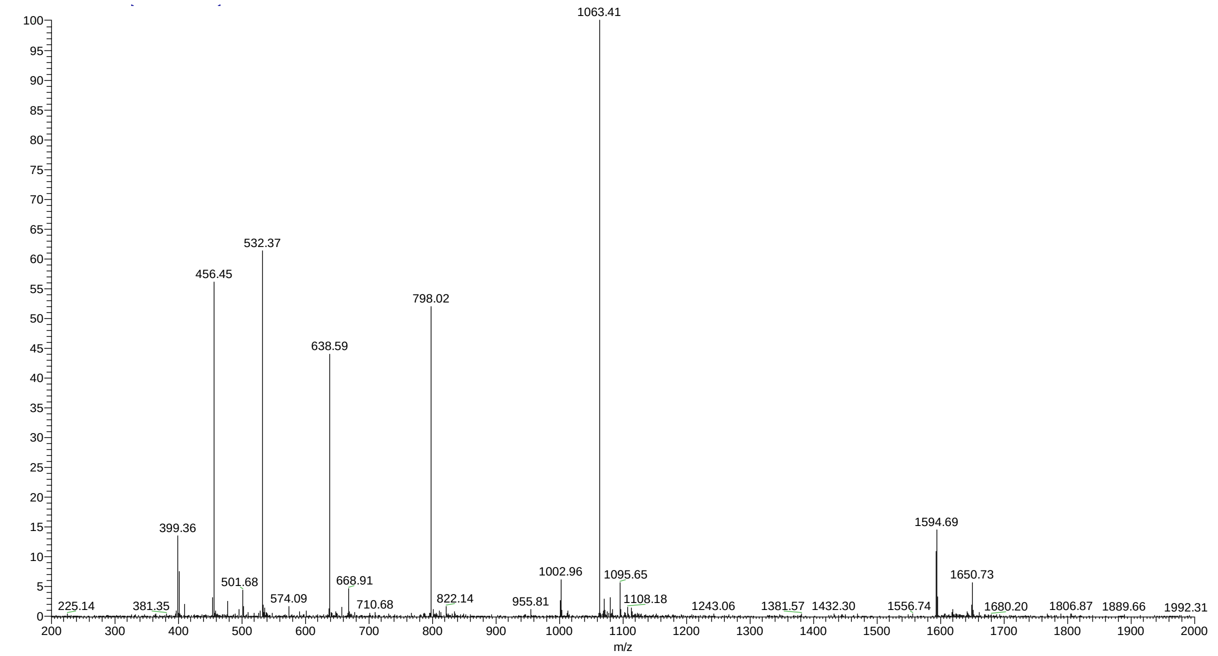

Penetratin-GG-WYKYW (RQIKIWFQNRRMKWKKGGWYKYW-NH_2)

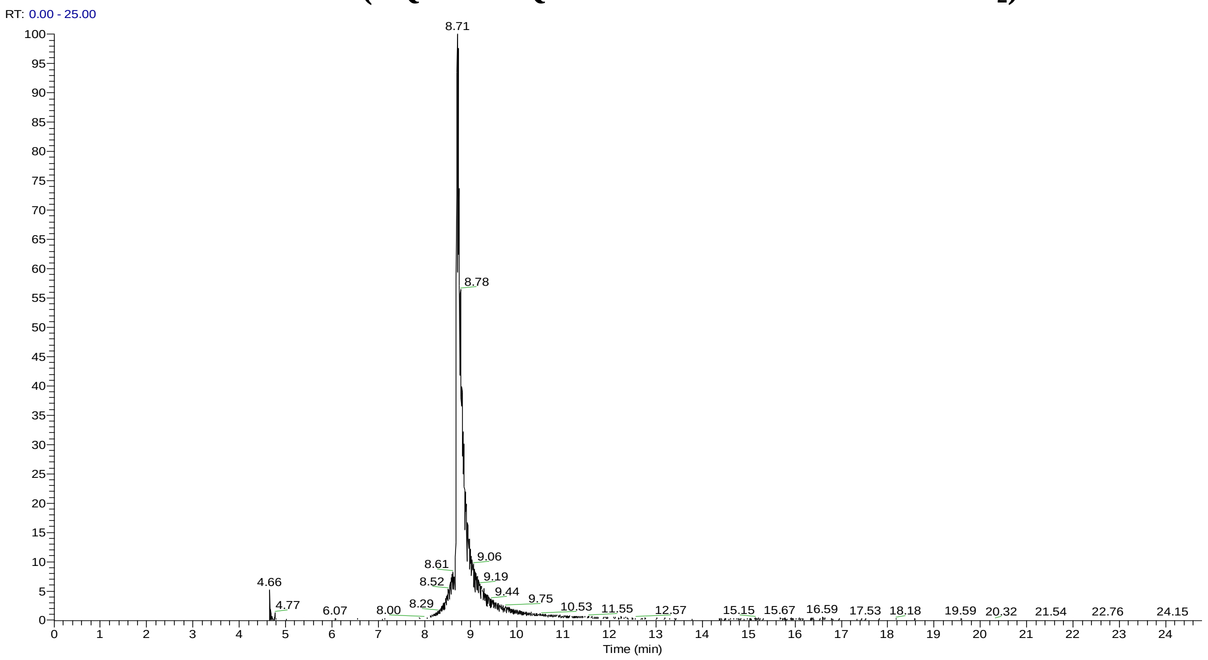

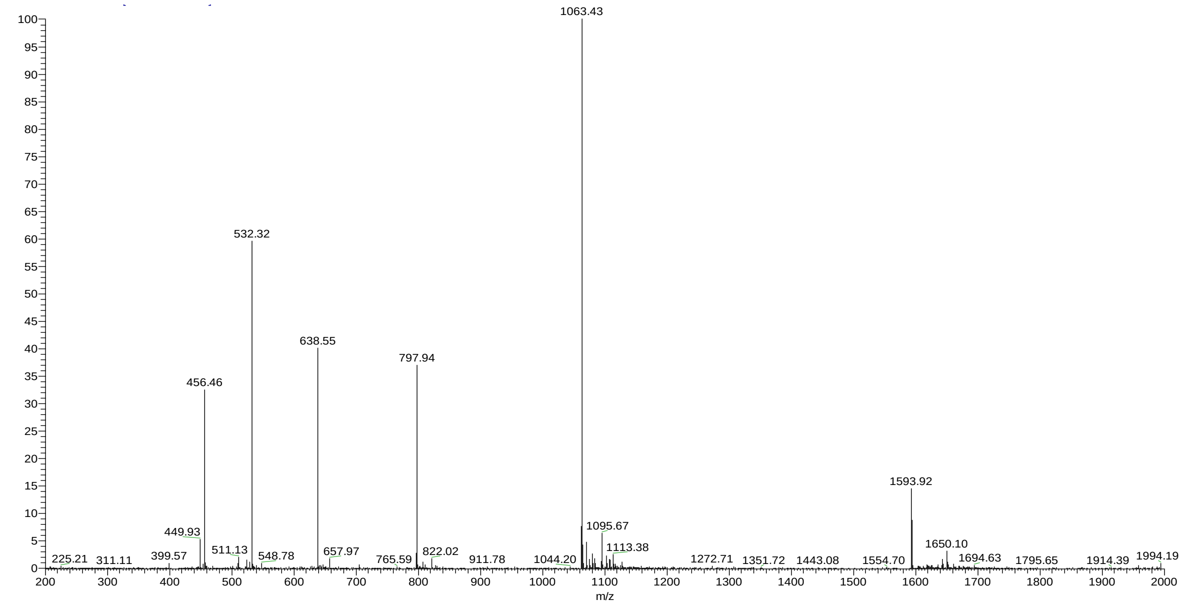

CFU-WYKYW-GG-Penetratin (5(6)-FAM-WYKYWGGRQIKIWFQNRRMKWKK-NH$_2$)

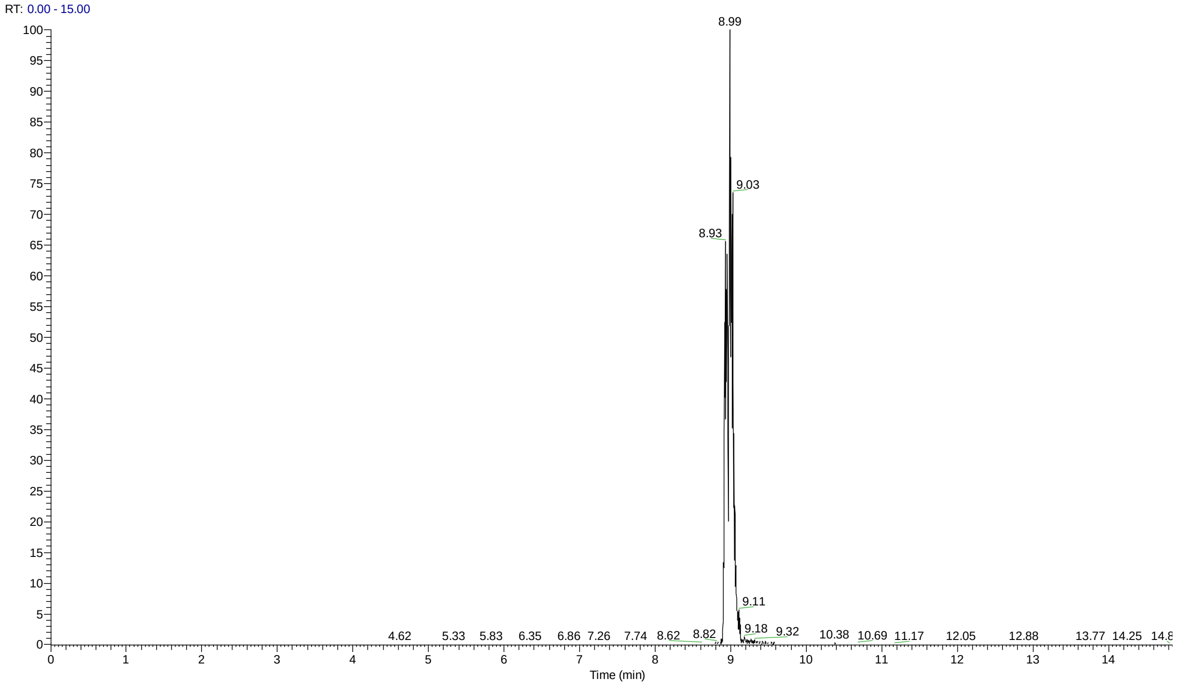

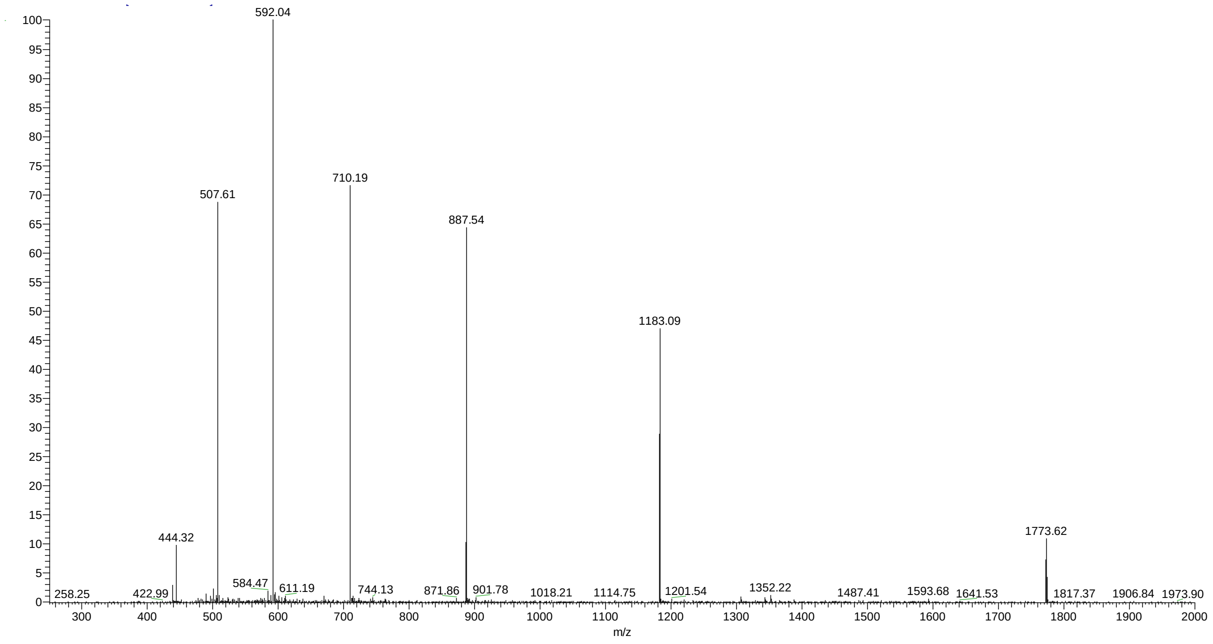

CFU-Penetratin-GG-WYKYW (5(6)-FAM-RQIKIWFQNRRMKWKKGGWYKYW-NH$_2$)

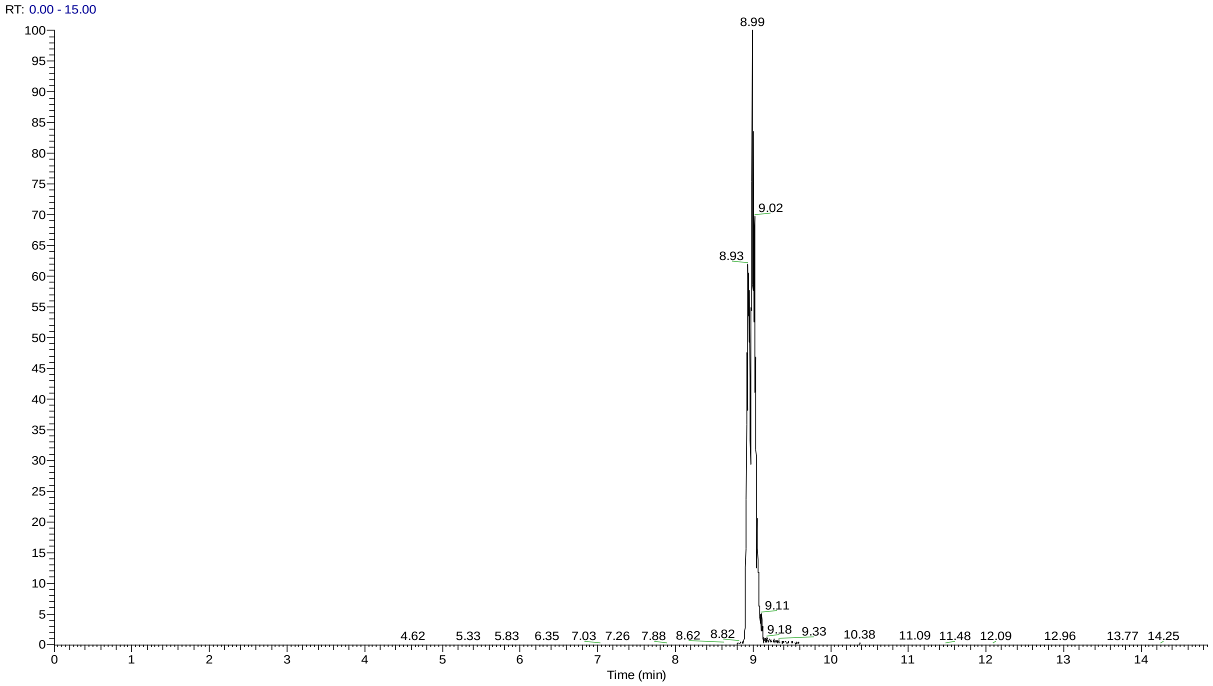

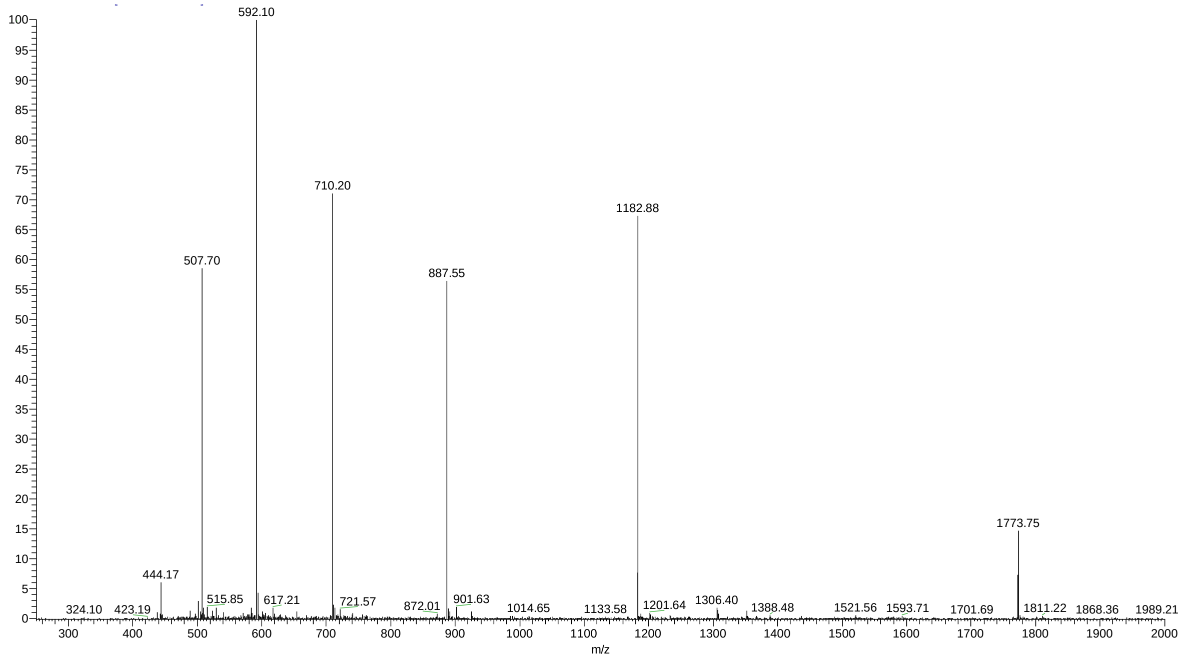

Biotin-Penetratin (K(Biotin)-RQIKIWFQNRRMKWKK-NH_2)

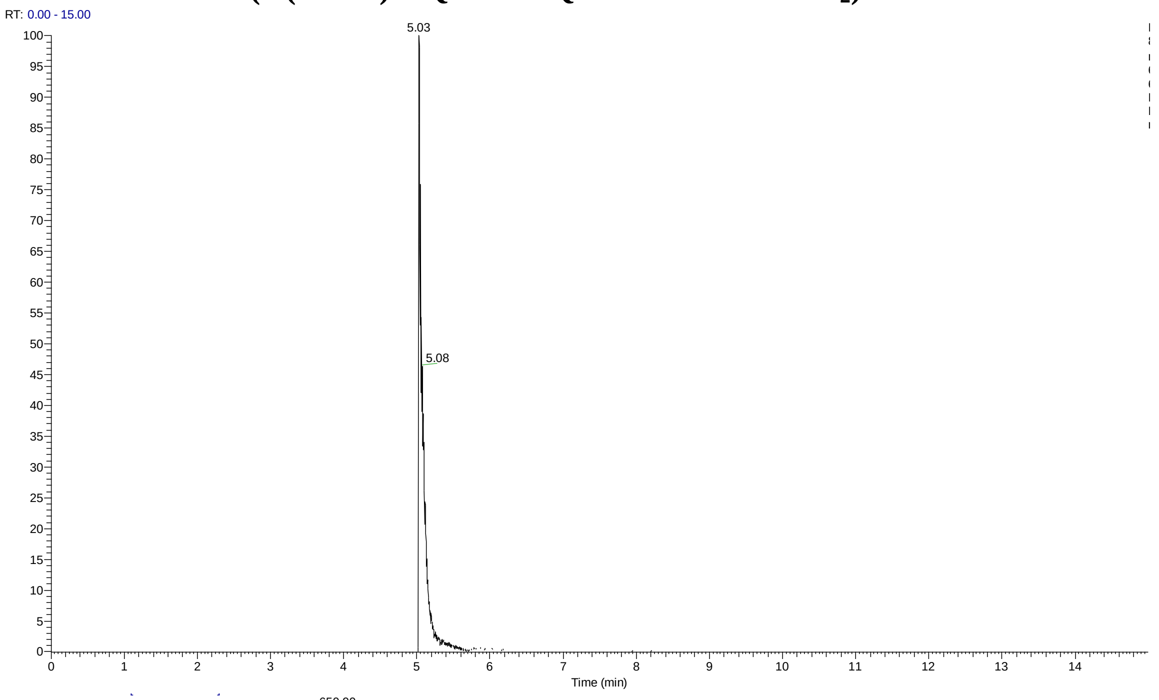

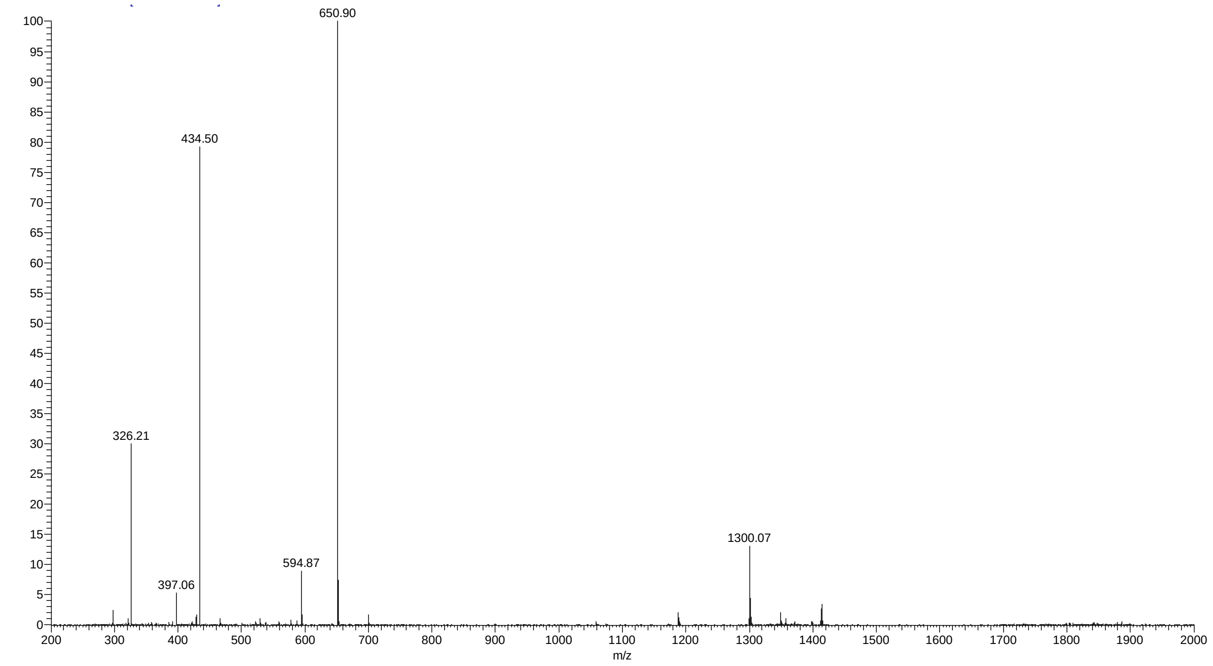

Biotin-Penetratin-GG-WYKYW (K(Biotin)-RQIKIWFQNRRMKWKKGGWYKYW-NH_2)

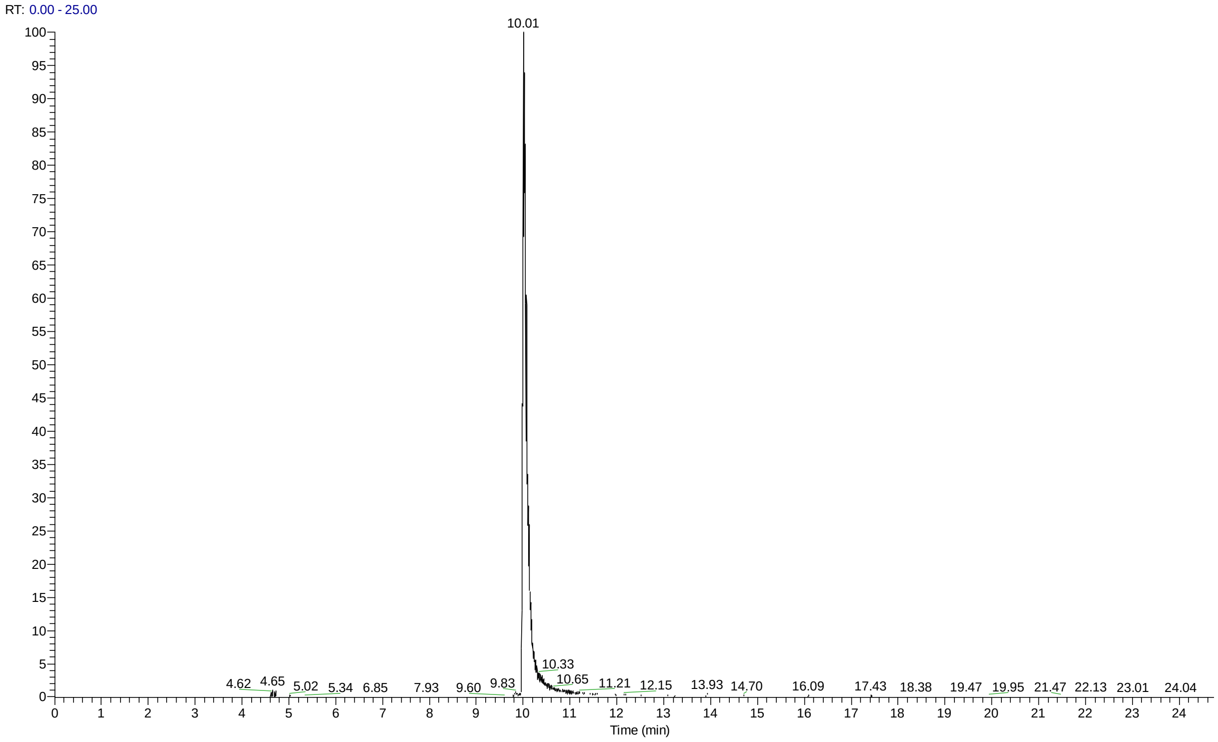

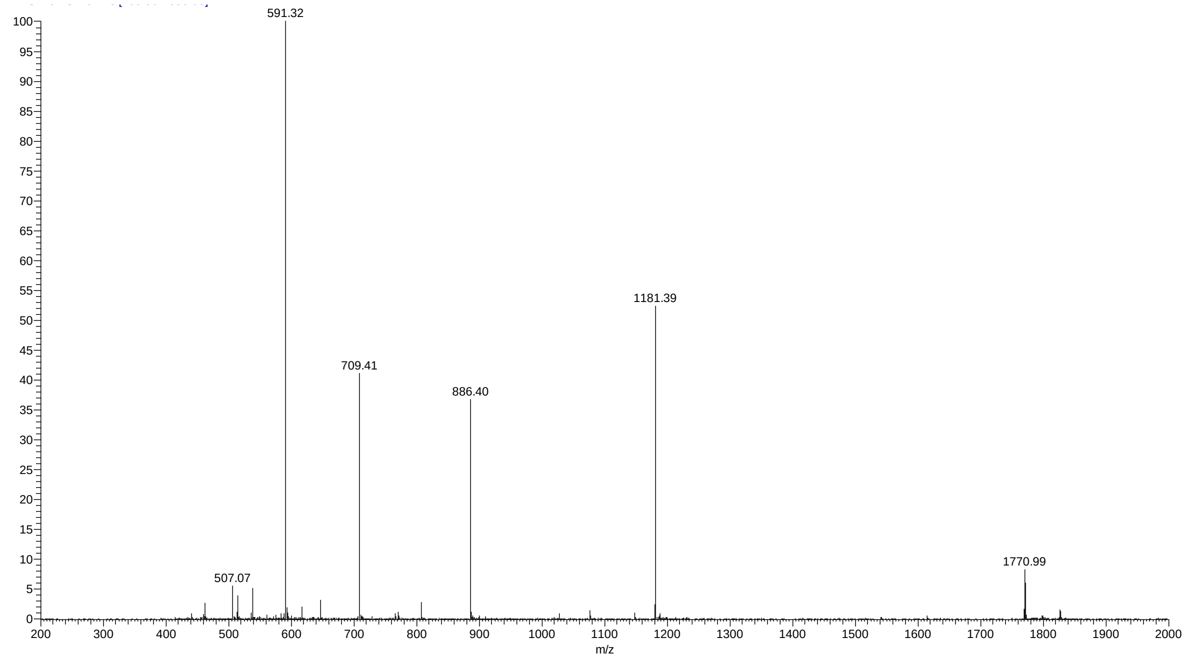

Biotin-PEG-WYKYW (K(Biotin)-(Ebes)$_3$-WYKYW-NH$_2$) (Ebes = N-(8-amino-3,6-dioxa-octyl)-succinamic acid

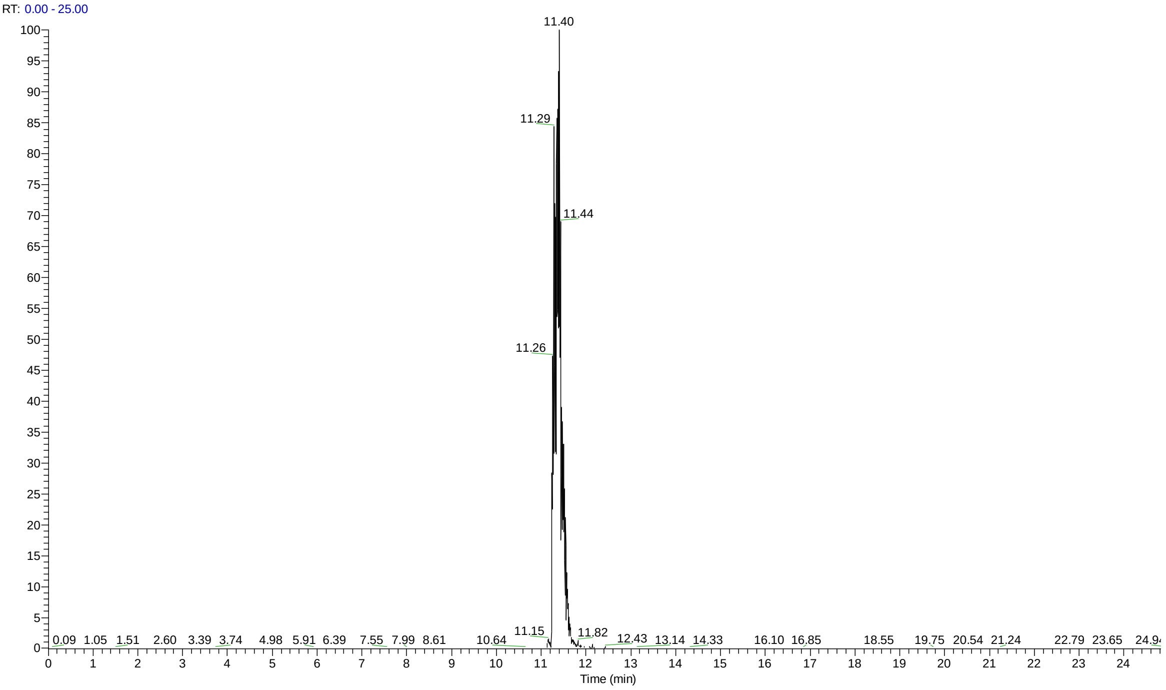

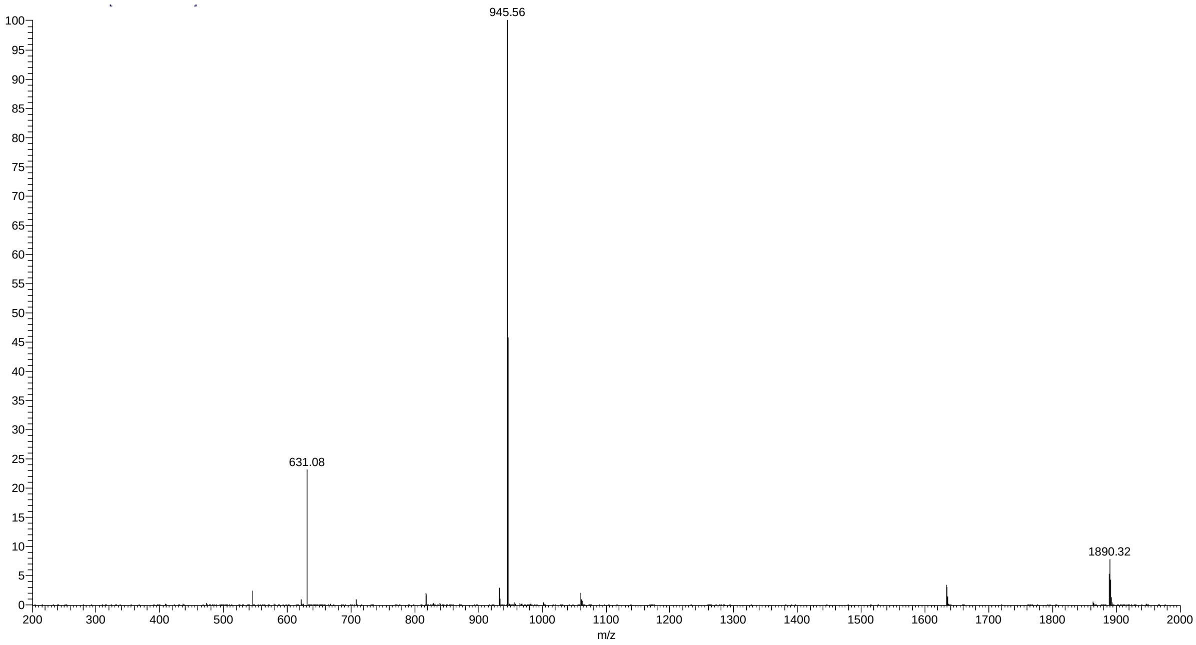

Supplementary Table 1. Theoretical and found masses of the sequences studied.

Sequence	Molecular weight		Expected m/z values (based on monoisotopic mass)		Detected m/z
	Average	Monoisotopic			
WYKYW	843.964	843.396	$[M+1H]^{1+}$	844.4	844.46
			$[M+2H]^{2+}$	422.71	422.93
CFU-WYKYW	1202.284	1201.716	$[M+1H]^{1+}$	1202.72	1202.44
			$[M+2H]^{2+}$	601.87	602.07
CFU-Penetratin	2604.072	2602.615	$[M+2H]^{2+}$	1302.31	1302.67
			$[M+3H]^{3+}$	868.55	869.26
			$[M+4H]^{4+}$	651.66	652.31
			$[M+5H]^{5+}$	521.53	522.05
			$[M+6H]^{6+}$	434.78	435.18
			$[M+7H]^{7+}$	372.81	373.1
WYKYW-GG-Penetratin	3186.805	3184.718	$[M+2H]^{2+}$	1593.37	1594.69
			$[M+3H]^{3+}$	1062.58	1063.41
			$[M+4H]^{4+}$	797.19	798.02
			$[M+5H]^{5+}$	637.95	638.59
			$[M+6H]^{6+}$	531.79	532.37
			$[M+7H]^{7+}$	455.97	456.45
			$[M+8H]^{8+}$	399.1	399.36
Penetratin-GG-WYKYW	3186.805	3184.718	$[M+2H]^{2+}$	1593.37	1593.92
			$[M+3H]^{3+}$	1062.58	1063.43
			$[M+4H]^{4+}$	797.19	797.94
			$[M+5H]^{5+}$	637.95	638.55
			$[M+6H]^{6+}$	531.79	532.32
			$[M+7H]^{7+}$	455.97	456.46
			$[M+8H]^{8+}$	399.1	399.57
CFU-WYKYW-GG-Penetratin	3545.125	3543.038	$[M+2H]^{2+}$	1772.53	1773.62
			$[M+3H]^{3+}$	1182.02	1183.09
			$[M+4H]^{4+}$	886.77	887.54
			$[M+5H]^{5+}$	709.61	710.19
			$[M+6H]^{6+}$	591.51	592.04
			$[M+7H]^{7+}$	507.16	507.61
			$[M+8H]^{8+}$	443.89	444.32
CFU-Penetratin-GG-WYKYW	3545.125	3543.038	$[M+2H]^{2+}$	1772.53	1773.75
			$[M+3H]^{3+}$	1182.02	1182.88
			$[M+4H]^{4+}$	886.77	887.55
			$[M+5H]^{5+}$	709.61	710.2

			**[M+6H]$^{6+}$**	591.51	592.1
			**[M+7H]$^{7+}$**	507.16	507.7
			**[M+8H]$^{8+}$**	443.89	444.17
Biotin-Penetratin	2600.246	2598.71	**[M+2H]$^{2+}$**	1300.36	1300.07
			**[M+4H]$^{4+}$**	650.68	650.9
			**[M+6H]$^{6+}$**	434.13	434.5
			**[M+8H]$^{8+}$**	325.85	326.21
Biotin-Penetratin-GG-WYKYW	3541.298	3539.133	**[M+2H]$^{2+}$**	1770.57	1770.99
			**[M+3H]$^{3+}$**	1180.72	1181.39
			**[M+4H]$^{4+}$**	885.79	886.4
			**[M+5H]$^{5+}$**	708.83	709.41
			**[M+6H]$^{6+}$**	590.86	591.32
			**[M+7H]$^{7+}$**	506.6	507.7
Biotin-PEG-WYKYW	1890.23	1889.96	**[M+1H]$^{1+}$**	1890.97	1890.32
			**[M+2H]$^{2+}$**	945.99	945.56
			**[M+3H]$^{3+}$**	630.99	631.08

Full STD spectra obtained with on- and off-resonance irradiations

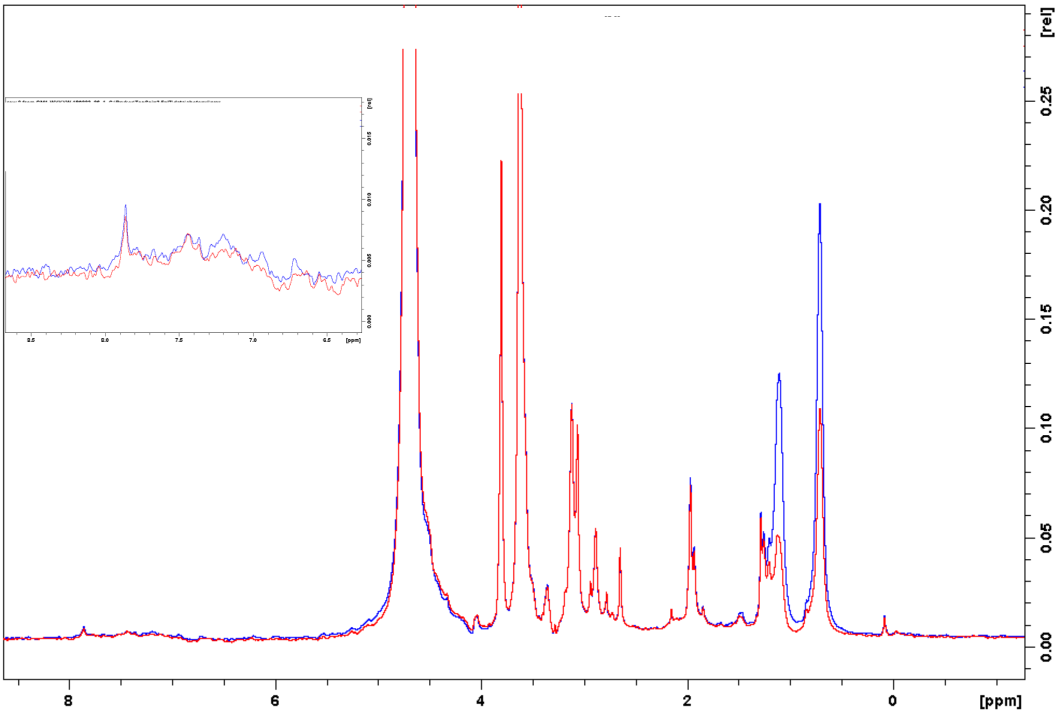

^{1}H STD of 20 μM WYKYW + 100 μM DPC + 20 μM ganglioside GM1 blue – on resonance, red off-resonance (enlarged amid regio in inset).

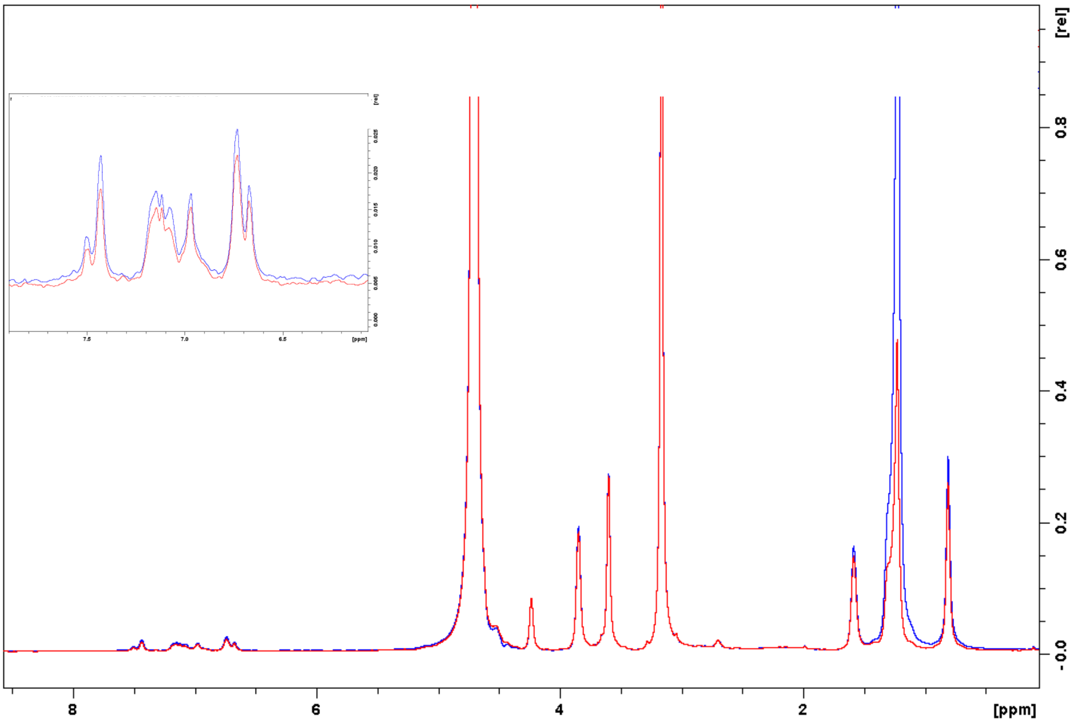

^{1}H STD of 20 μμ WYKYW + 100 μμ DPC + 20 μμ ganglioside GM3 blue – on resonance, red off-resonance (enlarged amid regio in inset).

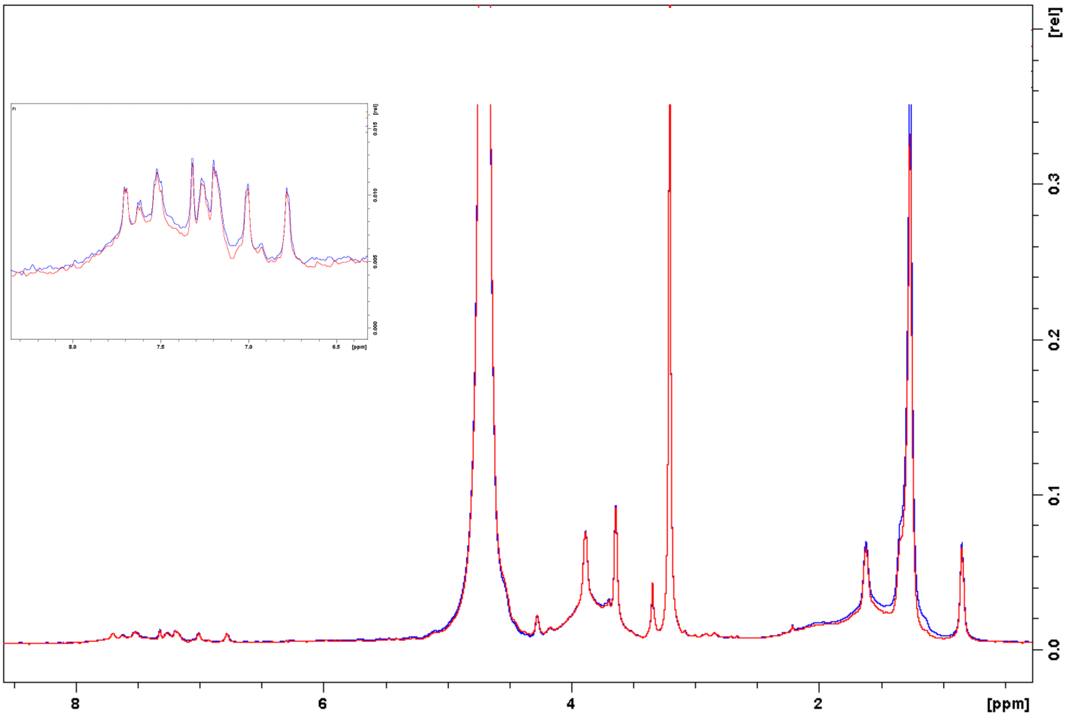

[1]H STD of 20 µµ WYKYW + 100 µµ DPC + 20 µµ ganglioside asialo-GM1 blue – on resonance, red off-resonance (enlarged amid regio in inset).

Raw ITC data for Figure 2

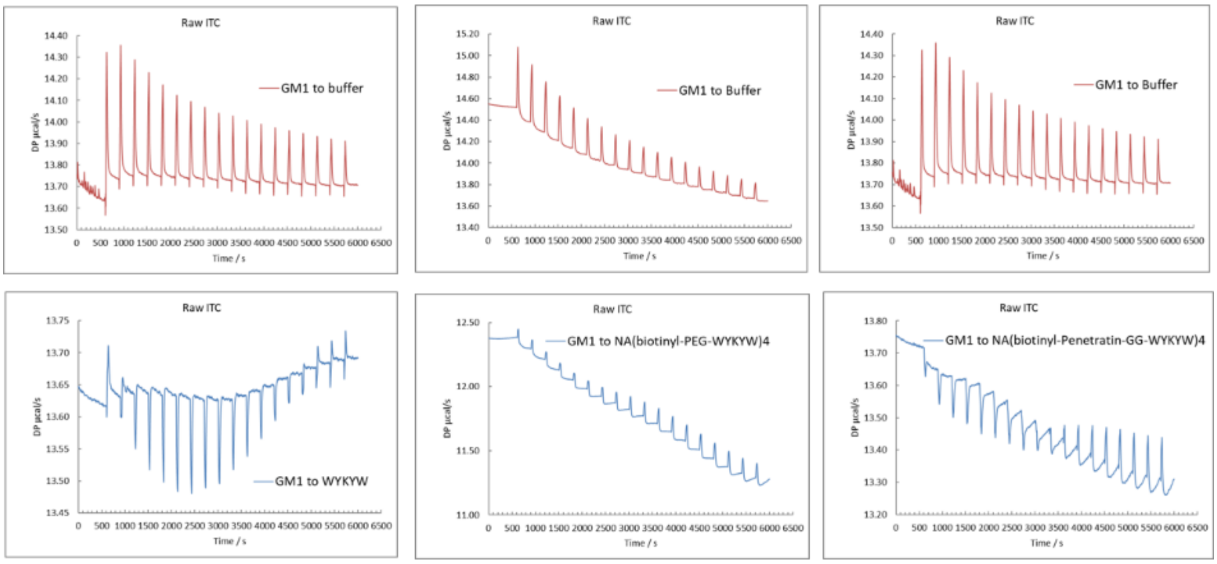

Raw ITC data for Figure S1

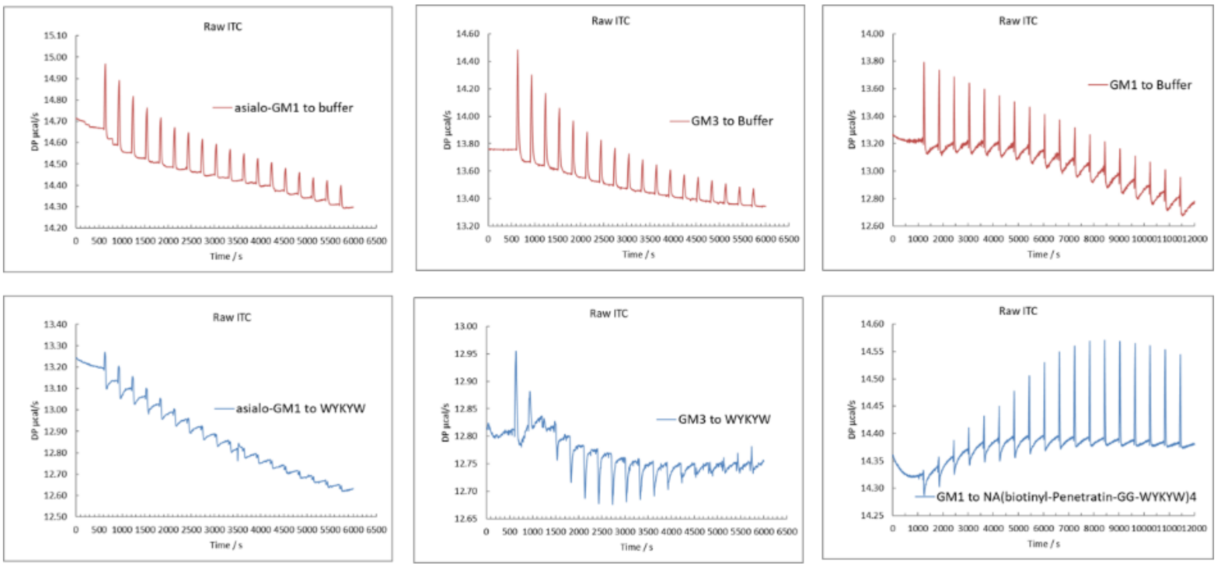

Raw ITC data for Figure S5

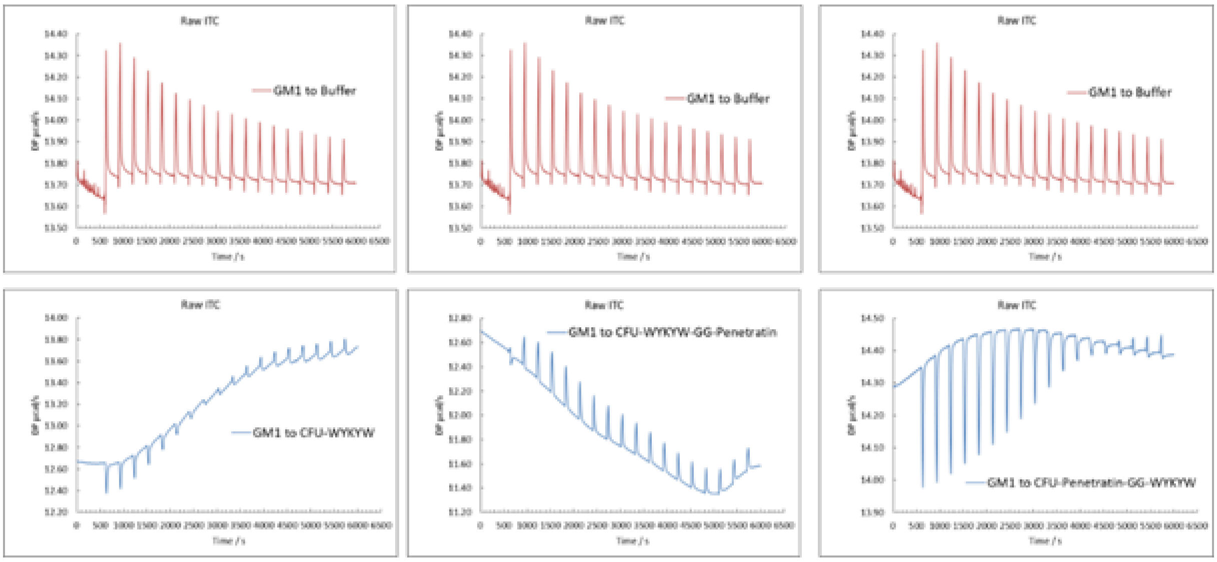

Supplement: Supplementary file 1 — Supporting Information [file ADVS-7-1902621-s001.pdf]
